# Dual-functional metalenses for the polarization-controlled generation of focalized vector beams in the telecom infrared

**DOI:** 10.1038/s41598-023-36865-z

**Published:** 2023-06-26

**Authors:** Andrea Vogliardi, Gianluca Ruffato, Simone Dal Zilio, Daniele Bonaldo, Filippo Romanato

**Affiliations:** 1 Department of Physics and Astronomy ‘G. Galilei’, University of Padova, Via Marzolo 8, 35131 Padua, Italy; 2 Padua Quantum Technologies Research Center, University of Padova, Via Gradenigo 6, 35127 Padua, Italy; 3 CNR-IOM Istituto Officina dei Materiali, S.S. 14-Km. 163,5, 34149 Trieste (TS), Italy

**Keywords:** Metamaterials, Nanophotonics and plasmonics

## Abstract

The availability of static tiny optical devices is mandatory to reduce the complexity of optical paths that typically use dynamic optical components and/or many standard elements for the generation of complex states of light, leading to unprecedented levels of miniaturization and compactness of optical systems. In particular, the design of flat and integrated optical elements capable of multiple vector beams generation with high resolution in the visible and infrared range is very attractive in many fields, from life science to information and communication technology. In this regard, we propose dual-functional transmission dielectric metalenses that act simultaneously on the dynamic and geometric phases in order to manipulate independently right-handed and left-handed circularly polarized states of light and generate focused vector beams in a compact and versatile way. In the specific, starting from the mathematical fundamentals for the compact generation of vector beams using dual-functional optical elements, we provide the numerical algorithms for the computation of metaoptics and apply those techniques to the design and fabrication of silicon metalenses which are able to generate and focus different vector beams in the telecom infrared, depending on the linear polarization state in input. This approach provides new integrated optics for applications in the fields of high-resolution microscopy, optical manipulation, and optical communications, both in the classical and single-photon regimes.

## Introduction

Since the seminal paper of Allen and co-workers in 1992^1^, beams carrying orbital angular momentum (OAM) have ignited a flourishing research area and inspired disruptive applications in a wide range of fields from life science to information and communication technology^2–4^. As a matter of fact, the peculiar intensity and phase distributions of OAM beams provide new optical tools for particle trapping and tweezing^5,6^, high-resolution imaging^7^, and lithography^8^, while leveraging OAM to encode information has become the key route to advance the capabilities of holographic devices^9^ and communication networks^10^, both at the classical and single-photon regimes, also in combination with other degrees of freedom, such as polarization. Non-separable combinations of well-defined spin and angular momentum states with opposite helicity, the so-called vector beams^11^, define 4-dimensional subspaces with intriguing properties and applications. Such families of beams constitute well-isolated mode groups for spatial division multiplexing in axially-symmetric optical fibres with step-index profiles^12,13^, and suggest an effective solution to expand the standard quantum-key distribution (QKD) protocols based on polarization. In fact, separable and non-separable combinations of spin and OAM define the two mutually-unbiased bases of a 4D QKD protocol with increased key rate and improved robustness and tolerance to noise, as demonstrated both in free-space^14,15^ and optical fiber^16^ applications. However, the technological transfer of such applications into real scenarios demands the combined control over the degrees of freedom of light to generate the required states using optical elements which are compact, versatile, and efficient, and furthermore foster the integration with today’s technology.

During the last 30 years, the ability to manipulate optical angular momentum has improved dramatically together with the capability to structure matter at the nanoscale and develop novel photonic devices^17–19^. The characteristic helical wavefronts of OAM beams inspired optical elements with a 3D staircase profile, i.e., spiral phase plates (SPPs)^20,21^, or their diffractive equivalent, the so-called fork holograms^22^. Such optical elements have been realized with liquid–crystal devices or high-resolution lithographic techniques^23^, proving the generation of OAM beams with high purity, while additional radial discontinuities^24,25^ enabled the control also of the radial number for the generation of higher-order OAM modes. The metasurface paradigm^26^ inflamed the evolution of SPPs from 3D surface relief structures to their 2D counterparts, the so-called *q*-plates. Instead of modulating the optical path with a spatially-variant thickness, *q*-plates implement an artificial material with fixed thickness and spatially-variant form birefringence, by exploiting the inherent anisotropy of liquid crystals^27–30^ or structuring matter in the form of 2D nano-resonators^31,32^ or subwavelength gratings^33^ using the well-established techniques of semiconductor manufacturing. The imparted phase is equal to twice the local angle formed by the extraordinary axis, while its sign depends on the handedness of the circular-polarization state in input^34^. The phase-conjugation mechanism opened to the generation of vector beams in a compact manner, by illuminating the optical element using linear polarization^35^. As a matter of fact, the two constituent circularly polarized states carry away OAM contributions of opposite signs, resulting into a vector beam with a polarization configuration which depends on the input polarization angle. However, the geometric phase approach limits the range of possible phase patterns which can be implemented. For instance, only beams with opposite OAM can be generated, while a lens term is not efficient, since only one of the two circular polarizations would experience focusing. Therefore, it would be extremely useful to decouple spin from phase reshaping in order to effectively extend the capabilities of geometric-phase optics.

The solution is given by the combination of both geometric and dynamic phase manipulation, by using anisotropic nano-resonators with both different rotations and shapes, respectively. Tailoring the cross-section it is possible to adjust the dynamic phases along the ordinary and extraordinary axes and therefore include a polarization-insensitive phase term. The proper combination with the polarization-dependent geometric phase enables one to encode two totally different optical operations for the two circular polarization states and design dual-functional metaoptics^36–38^.

In this work, we consider for the first time the design, fabrication, and test of dual-functional silicon metasurfaces for the generation of focalized vector beams in the telecom infrared. In particular, after providing a numerical recipe for the engineering of any dual-functional metaoptics, we apply that method to the design of silicon metasurfaces enabling the spin-controlled generation of focalized OAM beams at 1310 nm. The functionality of such devices extends straightforwardly to the generation of complex vector beams when illuminated under linear polarization. This study further enriches the portfolio of optical elements for complex beams generation, providing passive silicon optics with high efficiency and minimal footprint, for advanced applications in microscopy, optical micromanipulation, and classical and quantum information, with unprecedented levels of compactness, miniaturization, and integrability.

## Theory

### Vector beams bases

In this work, we suggest metaoptics which are able to generate different types of focused vector beams (VBs). VBs are solutions of Maxwell’s wave equation^1,39^ that can be represented as non-separable combinations of polarization states and spatial modes. Mathematically, this non-separable combination is described by introducing a 4-dimensional basis defined by the Cartesian product between the spatial mode basis and the polarization basis^40^.

We introduce the two-element basis $\left\{ {\left| R \right\rangle ,\left| L \right\rangle } \right\}$ of the polarization space, where $$\left| R \right\rangle = \left[ {\begin{array}{*{20}c} 1 & i \\ \end{array} } \right]^{T}$$ and $$\left| L \right\rangle = \left[ {\begin{array}{*{20}c} 1 & { - i} \\ \end{array} } \right]^{T}$$ represent a right-handed and left-handed circular polarization state, respectively (the normalization factor $1/\sqrt 2$ has been omitted), and the basis $\left\{ {LG_{0}^{ + 1} ,\;LG_{0}^{ - 1} } \right\}$ for the spatial mode space representing first-order Laguerre–Gaussian modes, carrying an azimuthal phase term $\exp ( \pm i\varphi )$. Then, the first order VBs are defined as linear combinations of the elements of the 4D basis:

1 $$\left\{ {LG_{0}^{ + 1} ,LG_{0}^{ - 1} } \right\} \otimes \left\{ {\left| R \right\rangle ,\left| L \right\rangle } \right\} = \left\{ {LG_{0}^{ + 1} \left| R \right\rangle ,LG_{0}^{ - 1} \left| L \right\rangle ,LG_{0}^{ + 1} \left| L \right\rangle ,LG_{0}^{ - 1} \left| R \right\rangle } \right\}.$$

Moreover, this 4D space can be represented as the direct sum of two 2D subspaces called Hybrid Poincaré Spheres (HPSs). These two HPSs include the vortex states (defined by the basis $\left\{ {u_{R}^{ + } ,u_{A}^{ + } } \right\}$) and the anti-vortex states (defined by the basis $\left\{ {u_{R}^{ - } ,u_{A}^{ - } } \right\}$), respectively^40^:

2 $$\left\{ {LG_{0}^{ + 1} ,LG_{0}^{ - 1} } \right\} \otimes \left\{ {\left| R \right\rangle ,\left| L \right\rangle } \right\} = \left\{ {u_{R}^{ + } ,u_{A}^{ + } } \right\} \oplus \left\{ {u_{R}^{ - } ,u_{A}^{ - } } \right\}$$

The general state of a first order vector beam can be described in terms of LG beams and circular polarization basis as:

3 $$W_{\theta ,\chi }^{ \pm } = \cos \left( \chi \right)e^{ - i\theta } LG_{0}^{ \pm 1} \left| L \right\rangle + \sin \left( \chi \right)e^{ + i\theta } LG_{0}^{ \mp 1} \left| R \right\rangle$$

where the two angles $\theta$ and $\chi$ refer to the coordinates of the corresponding point on the HPS^41^. Equation (3) suggests the generation of vector beams from the controlled superposition of two circularly-polarized beams with opposite spin and different spatial configurations using for instance a two-arm interferometric setup. By adding a half-wave plate in cascade, it is possible to switch the handedness of the circularly-polarized contributions and bounce between the two HPSs.

### Dual functional metalenses (DFML)

Here we propose a compact tool to generate vector beams by means of a single dielectric dual-functional metalens (DFML). A DFML is constituted of subwavelength units, the so-called metaatoms (MAs), arranged over a periodic lattice and acting locally as half-wave plates, in order to maximize the polarization conversion and, therefore, the optical efficiency^42–44^. In particular, by properly selecting a set of birefringent metaunits with different cross-sections, it is possible to enable the exploitation of both the Pancharatnam–Berry (geometric) phase and the dynamic one and allow a complex manipulation of the input beam as required for VB generation.

**Figure 1:**
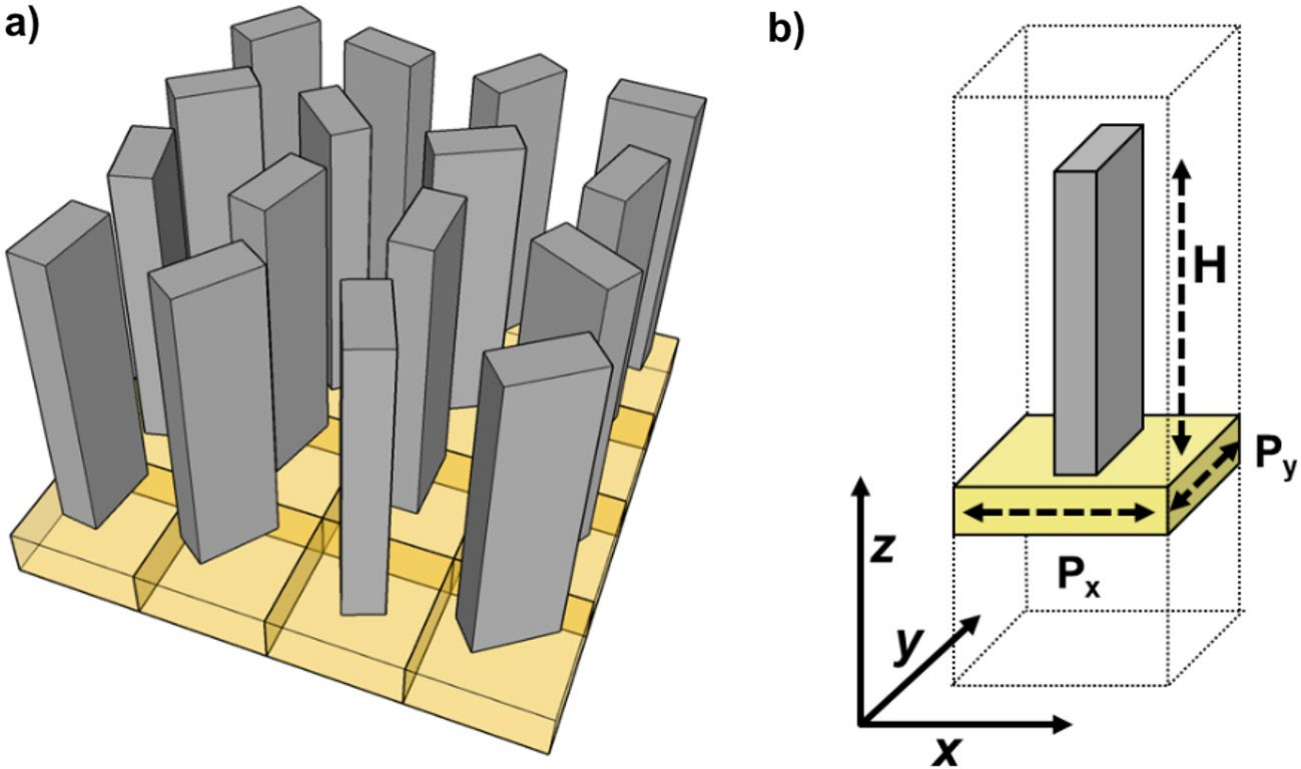
(**a**) Schematic representation of the metaatoms matrix composing a metalens. (**b**) 3D view of a metaatom. In this study, we impose *P*_*x*_ = *P*_*y*_ = 600 nm, and *H* = 850 nm.

**Figure 2:**
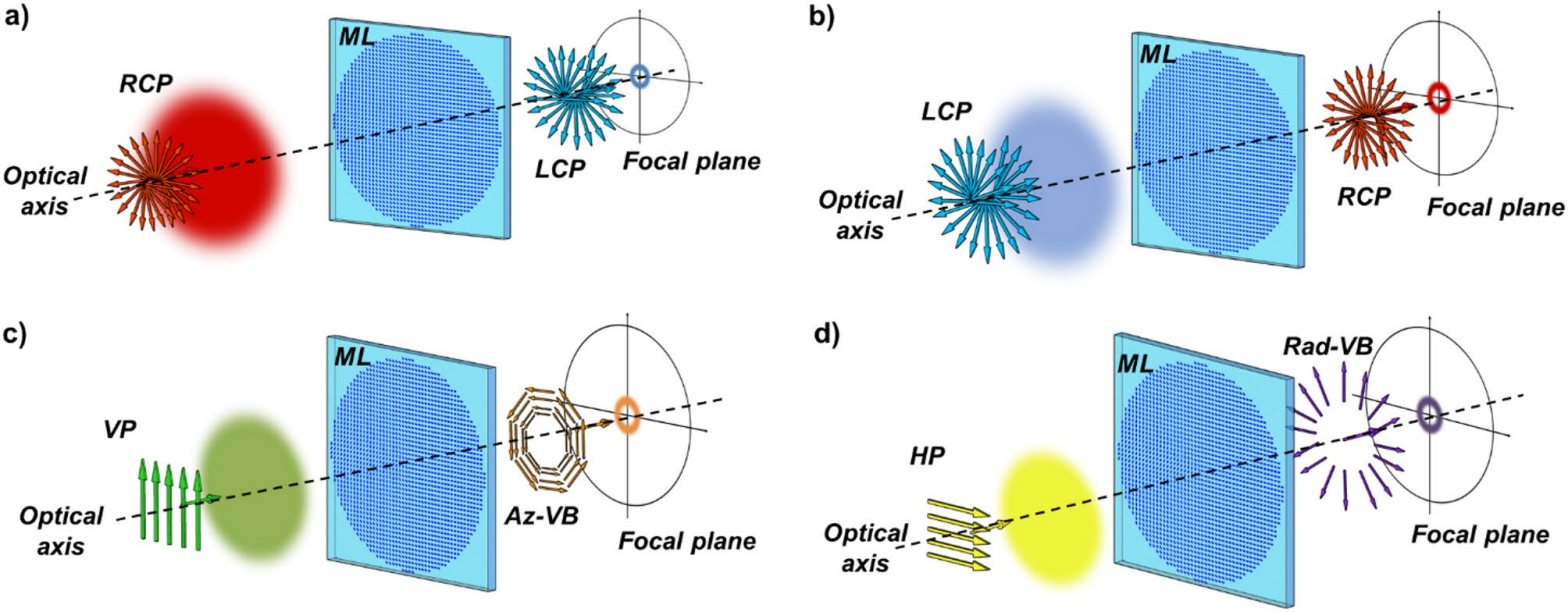
Dual Functional Metalenses paradigm for vector beams generation: using an impinging circularly polarized light (**a**,**b**), it is possible to generate focalized OAM beams with flipped helicity and opposite spin. Thus, for impinging linearly polarized light (**c**,**d**), it is possible to generate vector beams, such as the azimuthally polarized vector beam (Az-VB, **c**) or the radially polarized vector beam (Rad-VB, **d**).

Each nanopillar belongs to a library of nanostructures with different cross sections and orientations but the same height (Fig. 1a,b). Due to the subwavelength size of the metaunit, the input light experiences an effective medium with dynamic phase delays *δ*_x_ and *δ*_*y*_, referring to TM and TE linear polarizations, respectively. A rotation by *θ* of the anisotropic metaunit introduces a spin-dependent geometric phase equal to ± 2*θ,* depending on the handedness of the circular polarization state in input. By properly combining the control of both the geometric and dynamic phase, acting on the pillar orientation and varying its cross-section, respectively, the metalens is able to behave in two different ways depending on whether the input beam is right-handed (RCP) or left-handed circularly polarized (LCP) (Fig. 2a,b). As described in detail in^45^, the two different phase patterns *ϕ*^+^ and *ϕ*^*−*^ imparted to right-handed and left-handed circularly polarized beams are related to the dynamic and geometric phases by the following constitutive relations:

4 $$\delta_{x} = \frac{{\phi^{ + } (x,y) + \phi^{ - } (x,y)\,}}{2},$$

5 $$\delta_{y} = \frac{{\phi^{ + } (x,y) + \phi^{ - } (x,y)\,}}{2} + \pi ,$$

6 $$2\theta = \frac{{\phi^{ + } (x,y) - \phi^{ - } (x,y)\,}}{2}.$$

As a matter of fact, while the dynamic phase has the same effect both on LCP or RCP input beams, a change in the input polarization implies the transfer of symmetrical (opposite) geometric phases for the two circularly polarized states, that is:

7 $$J\left| L \right\rangle = - ie^{{i\left( {\delta_{x} + \delta_{y} } \right)/2}} e^{ + i2\theta } \left| R \right\rangle = e^{{i\phi^{ + } }} \left| R \right\rangle ,$$

8 $$J\left| R \right\rangle = - ie^{{i\left( {\delta_{x} + \delta_{y} } \right)/2}} e^{ - i2\theta } \left| L \right\rangle = e^{{i\phi^{ - } }} \left| L \right\rangle .$$

being *J* the Jones matrix of the anisotropic metaunit. Therefore, it is possible to obtain completely different behaviors under LCP or RCP impinging illumination by properly combining the two types of phases, that is, by controlling concurrently the shape of the pillars and their rotation point by point on the whole metasurface area^45–47^.

In this work, we exploit those properties to design and test DFMLs which are able to generate and focus different vector beams, depending on the linearly polarized state in input (Fig. 2c,d).

### Vector beams generation with DFML

As well known, it is possible to describe a linearly polarized beam as the linear combination of a left-handed circularly polarized state ($\left| L \right\rangle$) and a right-handed circularly polarized one ($\left| R \right\rangle$). More precisely, being $$\left| H \right\rangle = \left[ {\begin{array}{*{20}c} 1 & 0 \\ \end{array} } \right]^{T}$$ and $$\left| V \right\rangle = \left[ {\begin{array}{*{20}c} 0 & 1 \\ \end{array} } \right]^{T}$$ horizontal and vertical polarized states, respectively, and $$\left| \theta \right\rangle = \left[ {\begin{array}{*{20}c} {\cos \left( \theta \right)} & {\sin \left( \theta \right)} \\ \end{array} } \right]^{T}$$ a general linearly polarized state, the relationships between a linearly polarized state and its constituent circularly polarized contributions are: $\left| \theta \right\rangle = \left| R \right\rangle e^{i\theta } + \left| L \right\rangle e^{ - i\theta }$,$\left| H \right\rangle = \left| R \right\rangle + \left| L \right\rangle$ and $\left| V \right\rangle = i\left( {\left| R \right\rangle - \left| L \right\rangle } \right)$, where the normalization factor $1/\sqrt 2$ has been omitted.

For the sake of simplicity, we start considering a horizontally polarized beam in input, and we exploit the linearity of the Jones formalism. Therefore, for each metaatom we obtain:

9 $$J\left| H \right\rangle = J\left( {\left| R \right\rangle + \left| L \right\rangle } \right) = e^{{i\phi^{ - } }} \left| L \right\rangle + e^{{i\phi^{ + } }} \left| R \right\rangle ,$$

being *J* the Jones matrix of the DFML metaatom, *ϕ*^+^ and *ϕ*^*−*^ the phase patterns carried away by the right-handed and left-handed circularly polarized terms, respectively. As we are interested in generating focalized vector beams, we need to encode in the metasurface a phase profile which is able to transfer two focalized LG modes (with the same focal length but opposite OAM), corresponding to the basis of the spatial modes. To this purpose, we suggest a converging lens profile *ϕ*, being able to generate a focused beam carrying OAM at a desired position. In detail:

10 $$\phi (r,\varphi ) = \phi_{LG}^{\ell } + \phi_{{f,{{\varvec{\uprho}}}}} = \ell \varphi - \frac{2\pi }{\lambda }\left( {\sqrt {f^{2} + \left| {{\mathbf{r}} \cdot {{\varvec{\uprho}}}} \right|^{2} } - f} \right)$$

where $\ell$ is the amount of OAM per photon transferred to the impinging beam (or topological charge), in units of $\hbar$, $\lambda$ is the working wavelength, $f$ is the focal length, and ***ρ*** = ($x_{0}$,$y_{0}$) are the focus coordinates on the focal plane perpendicular to the propagation axis (*z*) and placed at a distance *f*. The first part of the equation, i.e., $\ell \varphi$, is the azimuthal phase that is necessary to generate an optical vortex with topological charge equal to $\ell$^47,48^, while the second part, i.e., $- 2\pi /\lambda \left( {\sqrt {f^{2} + \left| {{\mathbf{r}} \cdot {{\varvec{\uprho}}}} \right|^{2} } - \left| f \right|} \right)$, is a hyperboloid focusing profile $\phi_{{f,{{\varvec{\uprho}}}}}$, without spherical aberration under plane-wave incidence^49,50^, which is mandatory to focus the optical vortex onto a desired point in space. After imposing in Eq. (9)

11 $$\phi \pm = \phi_{LG}^{ \pm 1} + \phi_{{f,{{\varvec{\uprho}}}}} ,$$

we obtain:

12 $$J\left| H \right\rangle = e^{{i\phi_{{f,{{\varvec{\uprho}}}}} }} \left( {e^{{i\phi_{LG}^{ + 1} }} \left| L \right\rangle + e^{{i\phi_{LG}^{ - 1} }} \left| R \right\rangle } \right).$$

Recalling Eq. (3), it is worth noting that the second member is expected to generate, after free-space propagation, a radially-polarized vector beam defined as $W_{0,\pi /4} = LG_{0}^{ + 1} \left| L \right\rangle + LG_{0}^{ - 1} \left| R \right\rangle$ (the normalization factor $1/\sqrt 2$ has been omitted). The polarization-independent phase term $\exp (i\phi_{{f,{{\varvec{\uprho}}}}} )$ provides the required focusing in order to form the vector beam at the desired position in space.

Using the same designed DFML, but changing the impinging polarization from horizontal to vertical, i.e., $\left| V \right\rangle$, it is possible to generate another type of vortex vector beam focalized at the same point of the previous one. It is straightforward to prove that:

13 $$J\left| V \right\rangle = - ie^{{i\phi_{{f,{{\varvec{\uprho}}}}} }} \left( {e^{{i\phi_{LG}^{ + 1} }} \left| L \right\rangle - e^{{i\phi_{LG}^{ - 1} }} \left| R \right\rangle } \right),$$

which after propagation is expected to produce the VB with azimuthal polarization, focalized at a distance *f* and at a certain point ${{\varvec{\uprho}}} = (x_{0} ,y_{0} )$ after the metasurface.

Applying the same mathematical approach, but switching the phase profiles carried by the two circular polarizations, it is possible to generate also the associated anti-vortex vector beams. As a matter of fact, given the designed phase profiles $\phi \pm = \phi_{LG}^{ \mp 1} + \phi_{{f,{{\varvec{\uprho}}}}}$ it is straightforward to show that:

14 $$J\left| H \right\rangle = e^{{i\phi_{{f,{{\varvec{\uprho}}}}} }} \left( {e^{{i\phi_{LG}^{ + 1} }} \left| R \right\rangle + e^{{i\phi_{LG}^{ - 1} }} \left| L \right\rangle } \right),$$

15 $$J\left| V \right\rangle = - ie^{{i\phi_{{f,{{\varvec{\uprho}}}}} }} \left( {e^{{i\phi_{LG}^{ + 1} }} \left| R \right\rangle - e^{{i\phi_{LG}^{ - 1} }} \left| L \right\rangle } \right).$$

That proves the generation of focalized anti-vortex states associated with either a radial vortex state or an azimuthal vortex state, respectively, depending on the orientation of the impinging linear polarization.

It is worth noting that the same result can be achieved alternatively by flipping the chirality of the output polarization states, using for instance a half-wave plate in cascade, instead of changing the whole metasurface design.

With this design formalism, DFMLs do not work only under either vertically or horizontally polarized beams, but with any linear polarization state in input. In fact, imposing a general linear polarization state impinging on a DFML designed using Eqs. (12)–(13) (or Eq. (14)–(15)), it is straightforward to obtain the general formula:

16 $$J\left| \theta \right\rangle = e^{{i\phi_{{f,{{\varvec{\uprho}}}}} }} \left( {e^{{i\phi_{LG}^{ \pm 1} }} e^{ - i\theta } \left| L \right\rangle + e^{{i\phi_{LG}^{ \mp 1} }} e^{ + i\theta } \left| R \right\rangle } \right)$$

As shown in the Supplementary Information file, section S1, the same approach can be extended to the design of dual-functional metalenses for the generation of more complex beams with higher OAM values and non-null radial index. Under the illumination with linear polarization states in input, high-order vector beams can be generated.

## Results

### Metalens design

**Figure 3:**
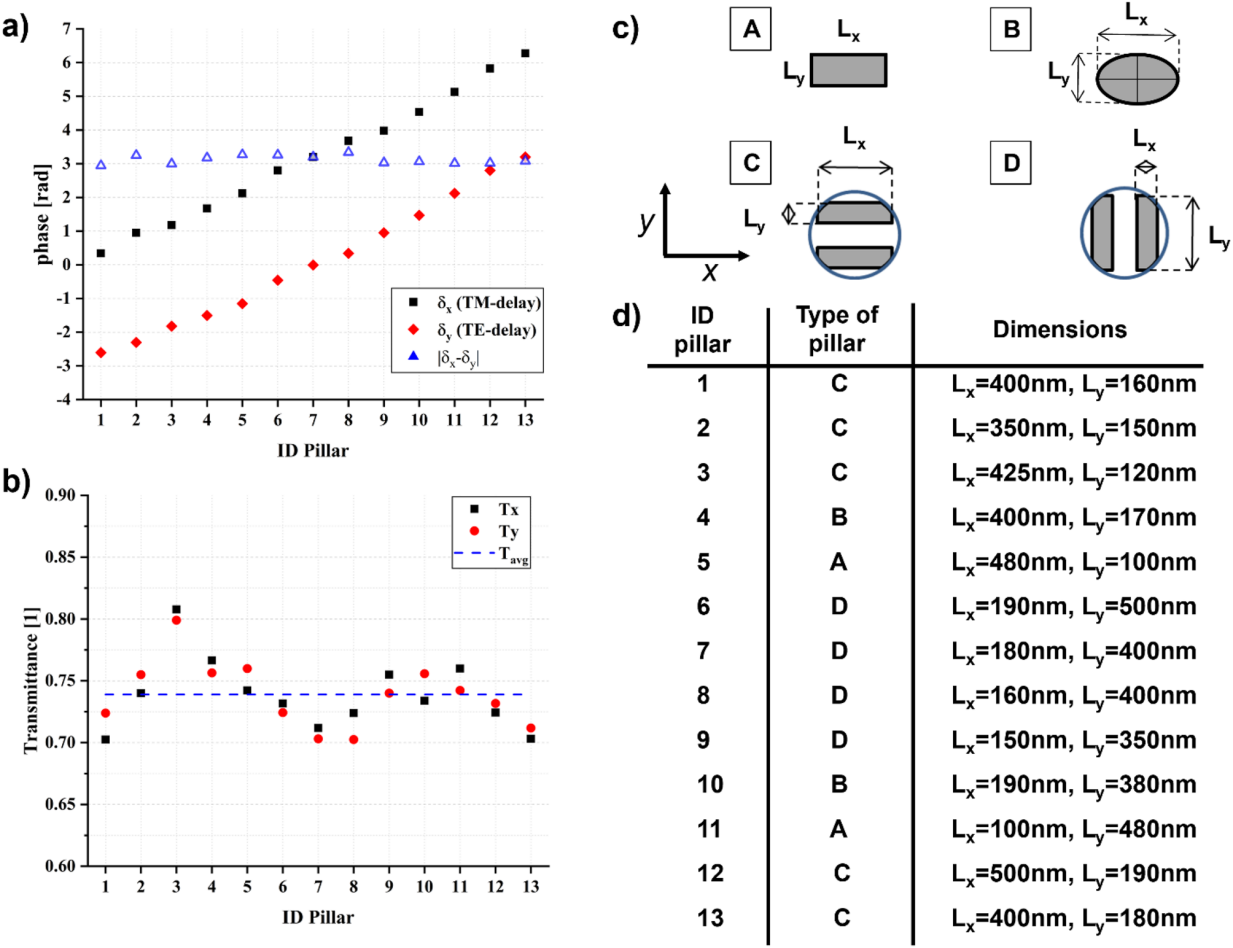
Library of different silicon nanopillars working at 1310 nm providing the recipe to build up a dual-functional metalens. (**a**) Phase delays for TE (*y*-delay) and TM (*x*-delay) polarizations, (**b**) Transmittance of each nanopillar under TE (*T*_*y*_) and TM (*T*_*x*_) polarization compared with the average transmittance (*T*_*avg*_) calculated among all the transmittance values. (**c**) Different types of pillars composing the meta-library. Rectangular and elliptical pillars (A–B) are the more common, with respect to the other two types (C–D) made of two paired pillars, with a rectangular base inscribed within a concentric circumference of radius 250 nm and placed at a distance of 300 nm between the two centers. (**d**) Complete list of the metaatoms library showing the type of pillars and the corresponding size.

To implement the wavefront engineering it is mandatory to find the set of optimized metaunits filling the whole metalens area. We performed custom finite element method (FEM) simulations (see “Methods”) to extrapolate the geometric features of each metaunit. Simulations were performed fixing the period of the metaatoms matrix at 600 nm along *x*-axis and *y*-axis (Fig. 1), and sweeping the sizes of the metaunit cross-section (*L*_*x*_, *L*_*y*_) (Fig. 3), considering fabrication constraints and the subwavelength regime, at the working wavelength of 1310 nm. Moreover, due to fabrication limitations, we imposed a fixed height *H* of 850 nm. Thus, for a fixed phase delay $\delta_{x}$ along the fast axis of the pillars, we selected the cross sections satisfying the condition $\Delta = \pi$, being $\Delta = \delta_{y} - \delta_{x}$. In particular, to ensure the HWP behavior of each metaunit, we selected metaatoms having a maximum phase difference of 0.03 rad from the HWP condition, i.e., $\left| {\Delta_{simulated} - \Delta } \right| \le 0.03\,rad$. At the same time, to ensure the polarization conversion under circularly polarized light, we imposed strict conditions on the transmissions for TE and TM polarizations. More precisely, we fixed $\left| {T_{x,i} - T_{y,i} } \right| \le 0.05$, being *T*_*x,i*_ and *T*_*y,i*_ the transmittance of the *i*-th metaatom for TM and TE polarizations, respectively. Concurrently, to guarantee a homogeneous transmittance over the whole metalens, we impose a maximum difference of 0.1 in transmittance among the *N* different metaatoms $\left| {T_{avg,i} - T_{avg,j} } \right| \le 0.1$, $i,j = 1,2,...,N$, being $T_{avg,k} = \left( {T_{x,k} + T_{y,k} } \right)/2$*.*

As a consequence, the previous requirements limit significantly the choice of possible cross-sections for the given thickness and shape. Therefore, in order to increase the degrees of freedom to find an adequate set of nanostructures covering the whole 2π range, different shapes have to be considered, such as rectangular, elliptical, and pairs of inscribed rectangular silicon pillars. A meta-library of 13 different nanopillars has been extrapolated from the simulations, which permits to have a well distributed 13-level discretization of the phase over the whole range 0–2π (Fig. 3). Conversely, we assumed no discretization on the geometric phase. In such a way, for predetermined phase patterns $\phi^{ + }$ and $\phi^{ - }$, we were able to calculate the corresponding maps for the dynamic and geometric phases, which provide the tool to compute the metaatoms pattern of the desired DFML. While the geometric phase map gives the local orientation of the metaatom, the required dynamic phase delays allow one to select the corresponding cross-section referring to the lookup table in Fig. 3d^45^.

To implement a first-order vector beams basis and prove the above-described design theory, we recall Eqs. (10) and (11), and we impose the phase pattern to the metalens under the choice $\ell$ = 1, *x*_*0*_ = *y*_*0*_ = 0, and *f* = 500 µm.

### Metalens fabrication

Electron Beam Lithography (EBL) is the ideal technique to convert the optimized computational pattern into the physical sample^23,51^. The metalens patterning was achieved using an EBL system (Carl Zeiss Sigma 300, 30 keV beam voltage) on $\left\langle {100} \right\rangle$ silicon substrates, spin coated with a 100-nm PMMA (AR-P-671.02, 950 kg/mol, Allresist GmbH) resist film and baked for 5 min on a hot plate at 180 °C. The exposure dose with an average value of 314 µC/cm^2^ was finely optimized taking into account the proximity dose effect, by applying a homemade developed software for dose correction. Samples were developed in a 3:1 solution of isopropanol (IPA) and methyl isobutyl ketone (MIBK) for 30 s and then rinsed with IPA.

**Figure 4:**
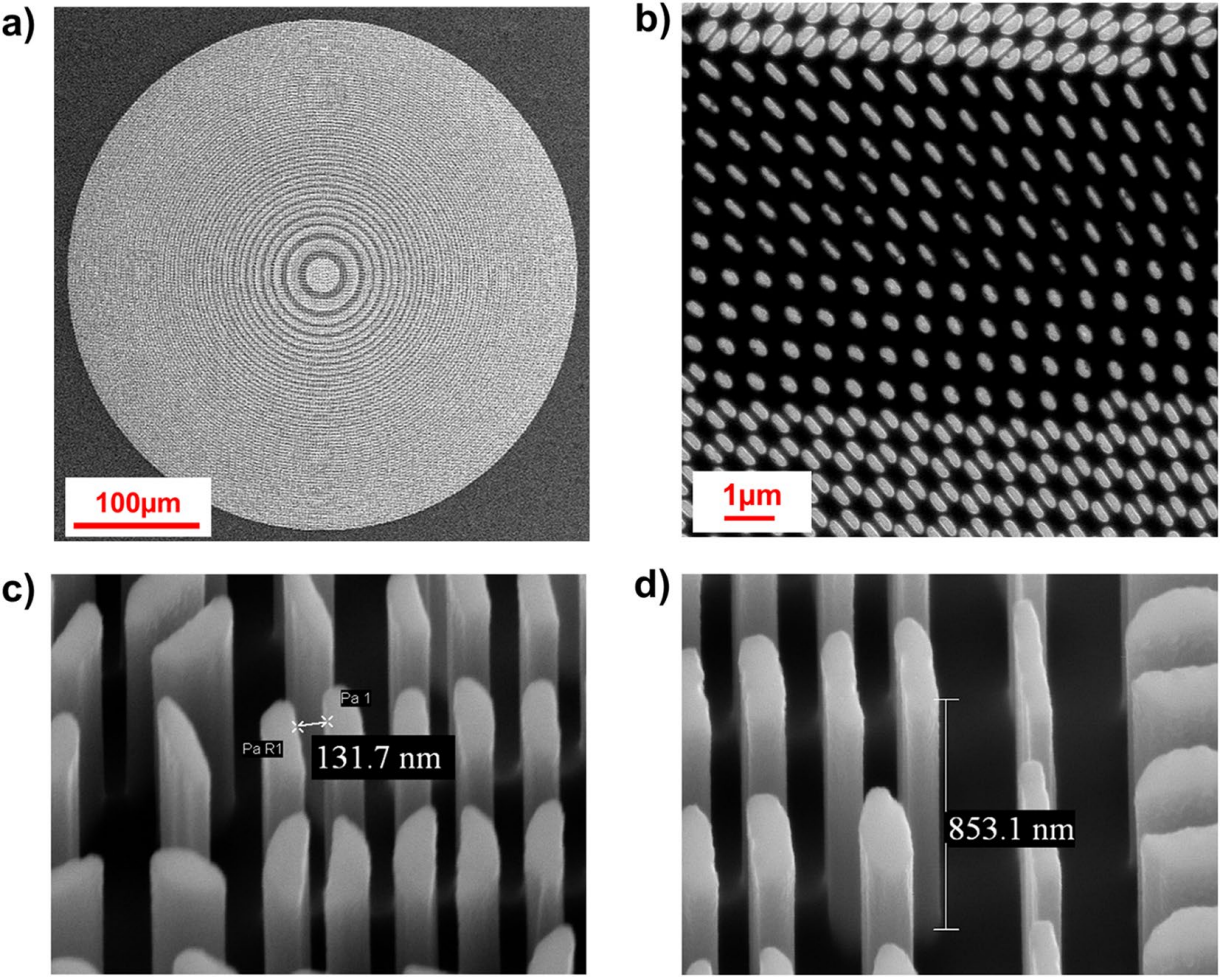
SEM images of the fabricated metalens. (**a**) Overall top-view of the entire metasurface. (**b**) Top view detail. (**c**,**d**) Details of the metasurface at higher magnification. The detailed images depict the high quality of the micromachined optics, confirming the correctness of pillars distribution and height size.

A thin Al_2_O_3_ film (12 nm) to be used as mask for the subsequent dry etching pattern transfer, was deposited by standard *e*-gun evaporation. Prior to deposition, the samples were treated by a controlled oxygen plasma cleaning process, to remove polymer residues on the exposed silicon region. After the lift-off of the PMMA resist in hot acetone (65 °C, 2 min in sonication), the pattern was transferred onto the underlying silicon substrate through Inductively Coupled Plasma Reactive Ion Etching (ICP-RIE). ICP-RIE was performed in a STS Multiplex ASE using a mixture of SF_6_:C_4_F_8_:Ar (3:6:1). Final characterization of the nanostructures (size, morphology, quality) was accomplished by scanning electron microscopy (SEM). In Fig. 4 some SEM images of the fabricated silicon metalens are shown.

### Optical simulations and characterization

**Figure 5:**
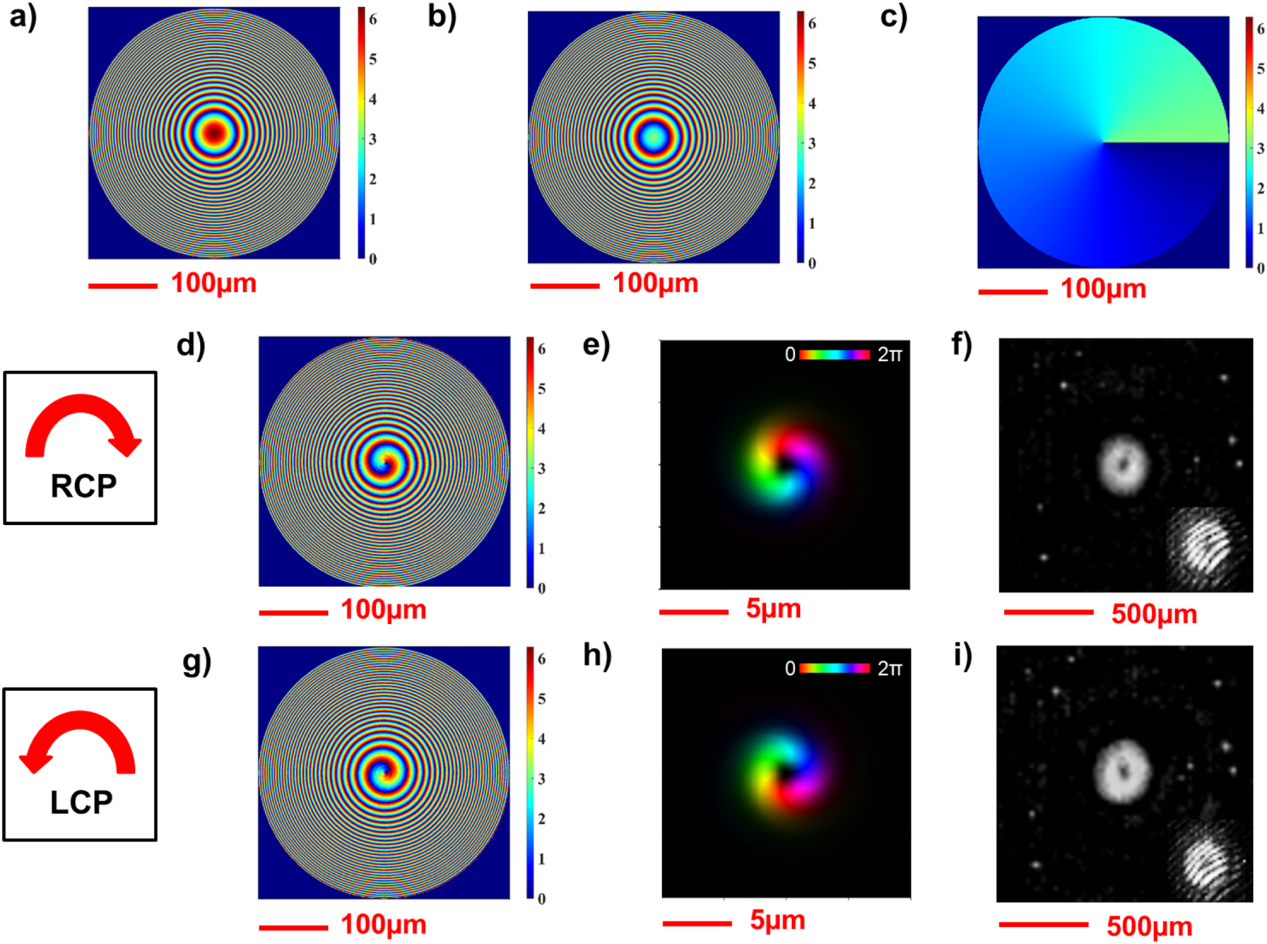
Design, simulation, and experimental output of DFML implementing Eq. (11). (**a**–**c**) ML design phase maps for *δ*_*x*_ (**a**), *δ*_*y*_ (**b**), and *θ* (**c**)^45^. (**d**,**g**) Show *ϕ*^+^ and *ϕ*^*−*^ phase maps, respectively, at 1310 nm. (**e**) Simulated intensity and phase of the propagated field under RCP polarization at *z* = 500 μm. (**h**) Simulated intensity and phase of the propagated field under LCP polarization at *z* = 500 μm. Brightness and colors refer to intensity and phase, respectively. (**f**,**i**) Measured intensity profiles of the generated structured beams with their associated interferograms. It is appreciable the different helicity in the OAM generation depending on the handedness of the circular polarization in input, since the two interferograms have opposite phase dislocations.

We start reporting the phase maps (Fig. 5a,b) of the fabricated metalens and the orientation of each metaunit (Fig. 5c) for the whole structure. Using a custom code (see “Methods”—“Optical simulations”), we simulated the optical response of the designed metaoptics to initially prove the ability to generate two structured beams with opposite OAM depending on the circular polarization state in input. The phase profiles experienced by the two circular polarization states are depicted in Fig. 5d,g and the simulated intensity and phase profiles of the structured beams at the focal plane are shown in Fig. 5e,h. As expected from the theory, two OAM beams are generated independently with opposite helicity, depending on the spin of the impinging beam. After that, using a custom characterization optical setup (see “Methods”—"Optical characterization”), we measured the intensity profiles and the interferograms of the generated beams (Fig. 5f,i). Two OAM beams with opposite helicity are clearly generated on the focal plane under either left-handed or right-handed circular polarization. From the interferograms, it is clearly observable that the two beams carry the same OAM with opposite helicity. In particular, a first-order OAM beam with a left-handed helicity is generated under RCP illumination, while under LCP illumination a first-order OAM with right-handed helicity is produced, which are the key elements to generate a focused vector beam.

**Figure 6:**
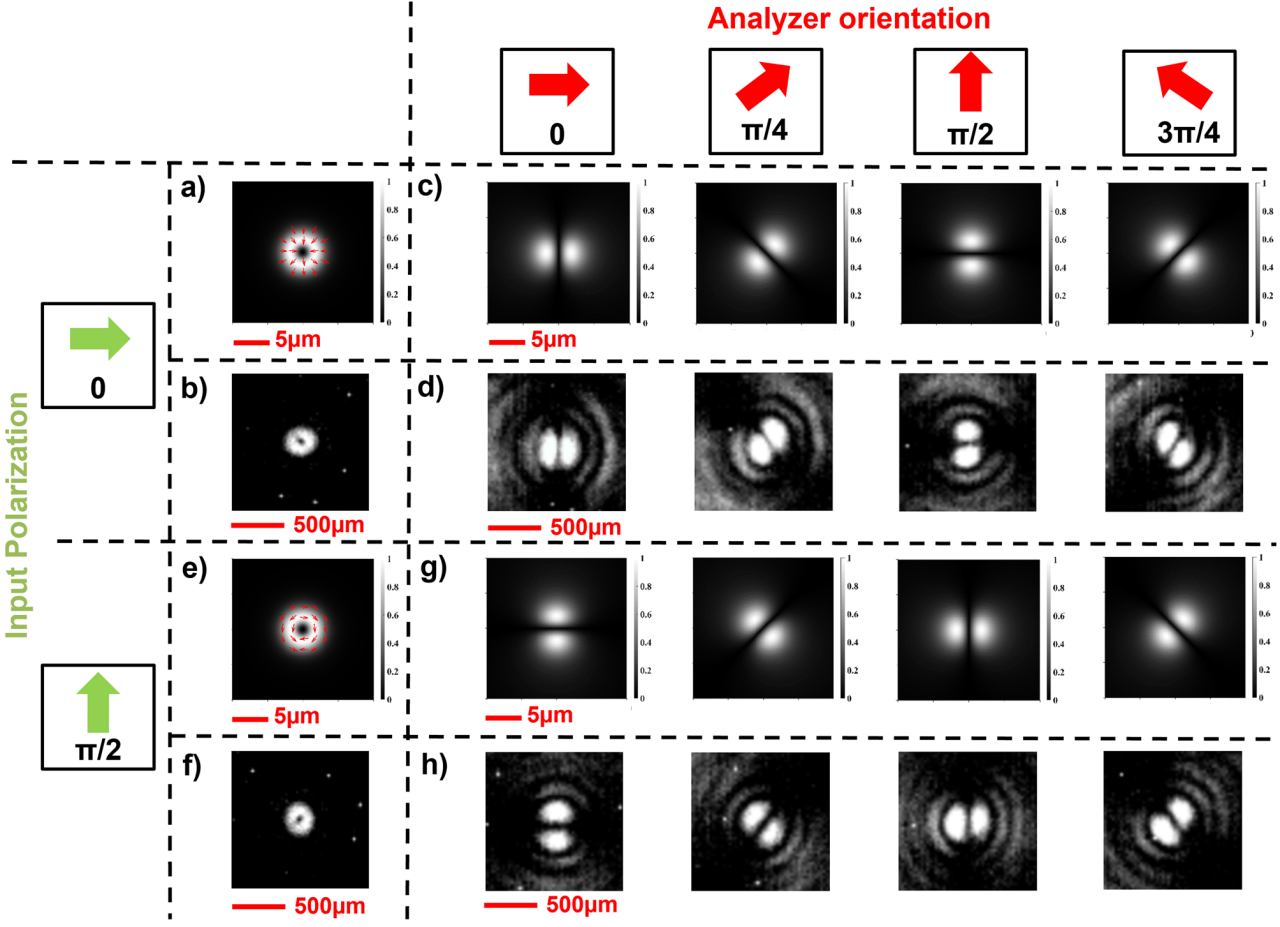
Generation of a vortex state basis following the design criteria of Eqs. (10) and (11), and analysis using a rotating linear polarizer (LP) in cascade. (**a**–**d**) Horizontally polarized light in input. (**a**) Simulated intensity profile and polarization plot of the generated radial VB. (**b**) Measured intensity profile of the generated radial VB. Simulated (**c**) and measured (**d**) intensity profiles of the radial vector beam analyzed with a rotating LP. (**e**–**h**) Vertically polarized light in input. (**e**) Simulated intensity profile and polarization plot of the generated azimuthal VB. (**f**) Measured intensity profile of the generated azimuthal VB. Simulated (**g**) and measured (**h**) intensity profiles of the azimuthal vector beam analyzed with a rotating LP.

After that, we verified the generation of vector beams using linearly polarized light in input. At first, we simulated the optical response of the metalens under either horizontal or vertical input linear polarization. Figure 6a,e exhibits the intensity profiles and the polarization states of the structured beams generated under the abovementioned conditions. It is clearly observable that under horizontal input polarization a radial vector beam is generated, rather than an azimuthal one when the impinging light is vertically polarized. Then, the intensity profile of the structured beam generated with our metasurface has been detected (Fig. 6b,f).

One of the easiest way to characterize the polarization pattern of a vector beam is to analyze it using a rotating linear polarizer. Filtering a first-order VB, a two-petal intensity structure (showing the constituent linearly-polarized Hermite–Gaussian beam) appears, with an orientation which depends on the type of vector beam. We started simulating the theoretical intensity profiles of a vector beam passing through an analyzer. The simulated profiles are shown in Fig. 6c,g and, as expected, associated with a counter-clockwise rotation of the analyzer there is a counter-clockwise rotation of the intensity profiles. Thus, we proved the goodness of the vortex state VBs generated by our metaoptics performing the same analysis experimentally. In particular, it can be noticed that the measured intensity profiles after the analyzer are in very good accordance with the simulated ones (Fig. 6d,h).

**Figure 7:**
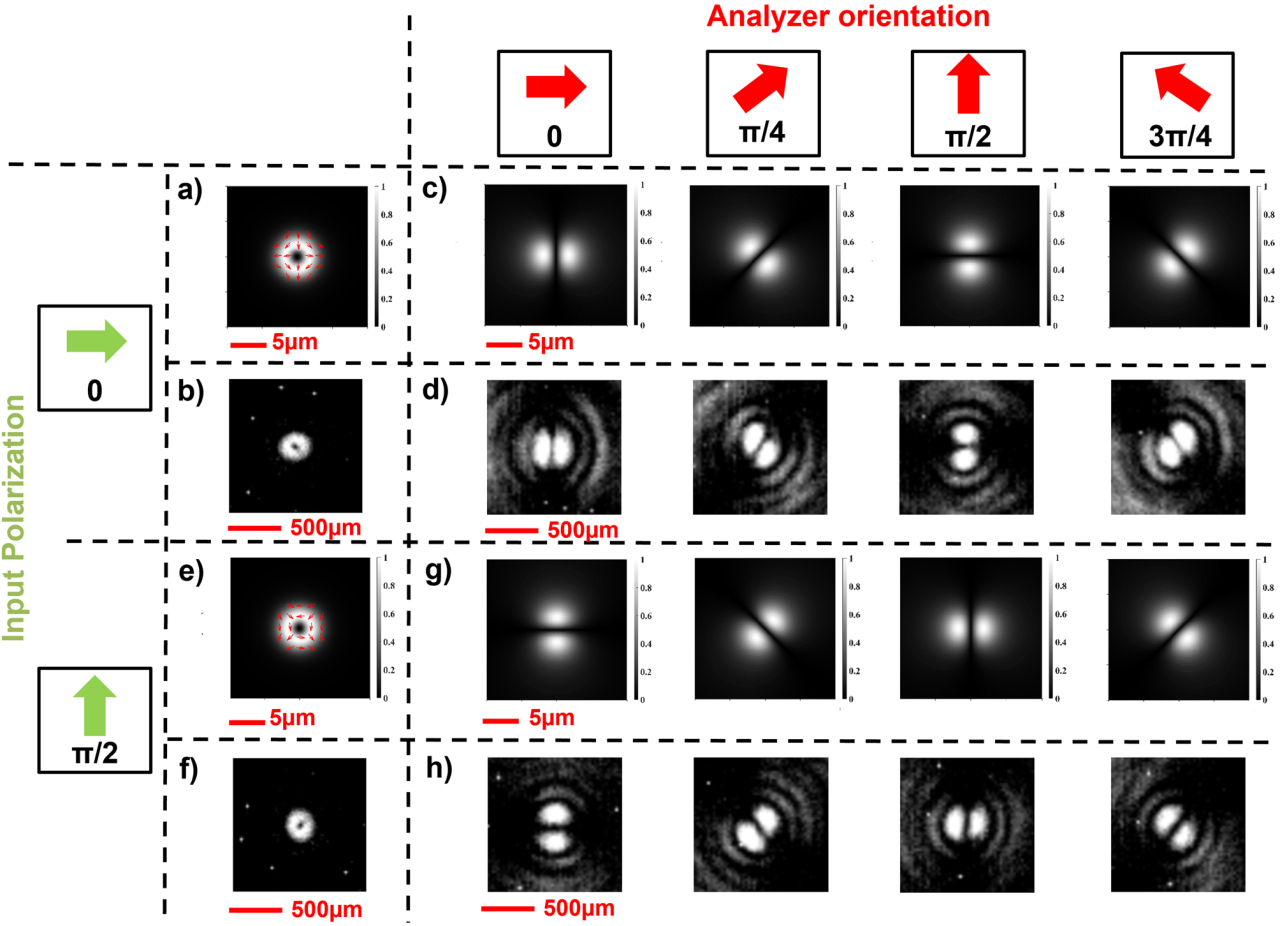
Generation of anti-vortex state basis and analysis using a rotating linear polarizer (LP). (**a**–**d**) Horizontally polarized light in input. (**a**) Simulated intensity profile and polarization plot of the generated anti-radial VB. (**b**) Measured intensity profile of the generated anti-radial VB. Simulated (**c**) and measured (**d**) intensity profiles of the anti-radial vector beam analyzed with a rotating LP. (**e**–**h**) Vertically polarized light in input. (**e**) Simulated intensity profile and polarization plot of the generated anti-azimuthal VB. (**f**) Measured intensity profile of the generated anti-azimuthal VB. Simulated (**g**) and measured (**h**) intensity profiles of the anti-azimuthal vector beam analyzed with a rotating LP.

In addition, we used the same designed metaoptics to generate focalized anti-vortex vector beams. By inserting a half-wave plate (HWP) after the DFML, we managed to flip the polarization handedness of the output beams moving from Eqs. (12), (13) to Eqs. (14), (15) as described in the “Theory” section. We performed the same simulations and characterizations described for the vortex states and we obtained the opposite behavior, as expected. In particular, it can be noticed that associated with a counter-clockwise rotation of the analyzer there is a clockwise rotation of the intensity profiles of the analyzed anti-vortex VB (Fig. 7).

## Discussion and conclusions

We have here presented the design of dual-functional silicon metalenses for the polarization-controlled generation of focused vector beams in the telecom infrared. The designed optical elements have been engineered to generate different vector beams depending on the polarization state of the impinging light. That is achieved by exploiting the intrinsic property of dual functional-metalenses to act both on the dynamic and geometric phases imparted to the input beam in order to induce spin-decoupled functionalities. In particular, we proved that imposing accurately optimized sub-wavelength patterns, judiciously designed for the decoupled functionalities of the metalens, it is possible to generate different vector beam bases, focalized at the same fixed point in space, using the polarization state of the impinging light as control parameter. We performed FEM simulations to define a library of silicon metaatoms, acting as half-wave plates with different phase delays along two orthogonal optical axes at 1310 nm, which has been used as a look-up table for the phase pattern generation of the proposed metasurface. The metalens has been fabricated using a two-step process: nanofabrication of the resist mask with EBL, and pattern transfer to the silicon substrate using inductively coupled plasma–reactive ion etching. In particular, the plasma conditions and etching time had to be properly optimized in order to reach the optimal thickness. However, as shown in the Supplementary Information file, section S2, the simulated optical response exhibits a good tolerance to deviations from the optimal thickness, exhibiting high values of efficiency conversion and transmission over a thickness range between 800 and 900 nm, that is, for a deviation from the design thickness up to 50 nm, which is far greater than the accuracy of the etching technique. In principle, the average transmittance around 75% can be improved further by using a low refractive index substrate, e.g., a glass slide, instead of silicon. For instance, a thin layer of amorphous silicon (*a*-Si) could be sputtered and patterned using the same two-step protocol (EBL + RIE). However, due to the different refractive index, a new run of FEM simulations must be conducted in order to find the new library of *a*-Si pillars satisfying the design constraints at 1310 nm. With respect to the usage of a *c*-Si substrate, the lower refractive index change at the different interfaces is expected to reduce the back reflection and therefore improve the overall transmittance. In addition, a proper anti-reflection coating could be deposited on the last substrate/air interface (not considered in this study).

Finally, we have proved the effectiveness of the metaoptics by characterizing the optical response under different impinging polarization states of light. The experimental results are in good agreement with the simulations, proving the correctness of both the design and the optimization of the fabrication protocols. While in the present paper we limited the study to the generation of the first-order vector modes, the method can be extended straightforwardly to the design of dual-functional metaoptics for the generation of high-order vector beams (see Supplementary Information file, section S1). In particular, the optimized set of pillars in Fig. 3 and the abovementioned design and fabrication techniques can be extended to the realization of any type of dual-functional metaoptics aimed to impart two different given phase patterns to left-handed and right-handed circular polarizations. The library of silicon metaatoms has been calculated and optimized for the wavelength of 1310 nm, then it is expected to work perfectly at the design wavelength. However, as shown with numerical simulations in the Supplementary Information file, section S3, the designed metalens exhibits high values of efficiency conversion and transmission over the whole telecom O-band from 1260 to 1360 nm, centered at the design wavelength. By further engineering the design of the metaatoms, it could be possible to control also the group delay and group delay dispersion^52^ and compensate for chromatic aberration, achieving the same focal length independently of the input wavelength over a wide band.

The ability to generate vector beams in different configurations using a single metasurface and the combination with passive optical elements (polarizer and wave-plate) permits both to simplify dramatically the optical path complexity and to ease significantly the alignment compared to other solutions^53–58^. Moreover, the fabricated metaoptics provide an upgrade in terms of both quality of the output vector beams and capability to focalize the generated structured light, in comparison with the metasurfaces previously reported in the literature^59,60^. The possibility to overcome the limits of geometric-phase conjugation and include a polarization-insensitive focusing term enables the generation and focalization of complex states of light with high efficiency and minimal footprint, suggesting compact and integrated optical architectures for high-order modes coupling in optical fibres, advanced microscopy, optical micromanipulation, and light-matter interaction.

## Methods

### FEM simulations

**Figure 8:**
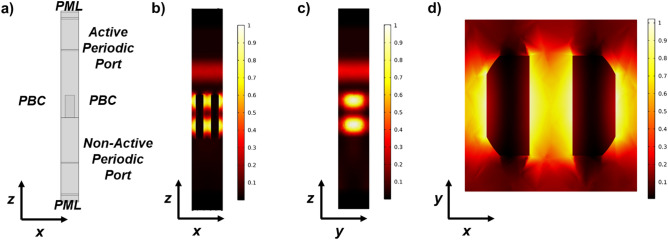
Example of FEM simulations of a metaunit (*P*_*x*_ = *P*_*y*_ = 600 nm) made of silicon nanopillars (*L*_*x*_ = 150 nm,* L*_*y*_ = 300 nm, and *H* = 850 nm, i.e., pillar #9 in Fig. 3) over a silicon substrate. (**a**) Boundary conditions imposed to properly simulate the nanostructure. *PBC* periodic boundary conditions. *PML* perfectly matched layers. (**b**–**d**) Electric field under TE polarization in input impinging from the air side. Lateral cross-sections at *y* = 0 (**b**), *x* = 0 (**c**), and top-view cross-section at *z* = *H*/4 (**d**). Input wavelength λ = 1310 nm. Colors refer to the intensity of the electric field (a.u.).

We set up custom-made Finite-Element Method (FEM) numerical simulations in the wavelength domain (using COMSOL Multiphysics^®^) to find the best set of metaatoms satisfying the DFMLs requirements described above (Fig. 8). Each subunit has been defined as a silicon nanopillar (*n*_*Si*_ = 3.5030) surrounded by air (*n*_*Air*_ = 1) placed on the top of a silicon substrate (*n*_*Si*_ = 3.5030). All the materials were considered as non-absorbing (*n* = *Re*(*n*), *Im*(*n*) = 0). Thus, we imposed some conditions to simulate properly the nanostructures: Periodic Port conditions were set in the substrate at a distance equal to $\lambda$ from the nanopillar and at a distance greater than $\lambda$ over the pillar, both to collect the scattering parameters of the structure and simultaneously ensure the far-field regime^61^; Perfectly Matched Layer (PML) conditions have been imposed outside the ports at a distance greater than $\lambda$ to visualize the transmitted and reflected fields, and to absorb the field over a certain distance to avoid unwanted multiple reflections. Finally, Periodic Boundary conditions (PBC) were set (along the *xz* and *yz* planes) to permit the correct simulation of the interaction between the various metaunits of the metalens (Fig. 8a)^62^. Among all the possible solutions, a subset of 13 pillars is selected covering the whole 2π range of dynamic phase and satisfying the conditions of half-wave plate and equal transmissions for TE and TM polarizations within the tolerance described above. Using custom scripts in MatLab, the maps of dynamic and geometric phases are converted into a GDSII file encoding the cross-sections of the metaatom, i.e., type 1–13 and orientation, in each cell of the metasurface 2D array with period of 600 nm in the *x-* and *y-*directions. Finally, then the CAD file is uploaded on the software of the electron-beam lithographic system and the design is materialized after exposure and development on the resist layer.

### Electron beam lithography

The metalens lithographic design was patterned using an Electron Beam Lithography system at 30 keV beam voltage on $\left\langle {100} \right\rangle$ silicon substrates, spin coated with a 100-nm PMMA (AllResist-P-671.02, 950 kg/mol) resist layer and baked for 5 min on a hot plate at 180 °C. The exposure dose at an average value of 314 µC/cm was finely optimized in order to compensate the proximity dose effect using a custom developed software. Samples were developed in a 3:1 solution of isopropanol (IPA) and methyl isobutyl ketone (MIBK) for 30 s and then rinsed with IPA. Then, the pattern was transferred from the resist to the underlying silicon through Inductively Coupled Plasma Reactive Ion Etching (ICP-RIE). An aluminum oxide hard mask has been deposited beforehand by e-gun evaporation and subsequent lift-off in hot acetone and sonication. ICP-RIE was performed in a STS Multiplex ASE with a plasma composition of 3 SF_6_: 6 C_4_F_8_: 1 Ar. Prior to deposition and etching, and following etching, oxygen plasma cleanings were executed in order to remove polymer residues and impurities. The quality and morphology of the nanostructures were assessed using scanning electron microscopy.

### Optical simulations

To simulate the optical response a custom MatLab^®^ code was used implementing the Fresnel propagator for a squared simulation window with a side of 400 µm and pixel size of 600 nm^63^. The simulations considered metalenses of radius 200 µm, designed at the working wavelength of 1310 nm with a phase discretization of 13 levels (Fig. 3) and illuminated by a Gaussian beam as given by ($\exp ( - (x^{2} + y^{2} )/w_{0}^{2} )$), with a beam waist ($w_{0}$) of 100 µm in order to cover adequately the metasurface area and avoid boundary effects. The intensity of the simulated electric field has been normalized to unity.

### Optical characterization

**Figure 9:**
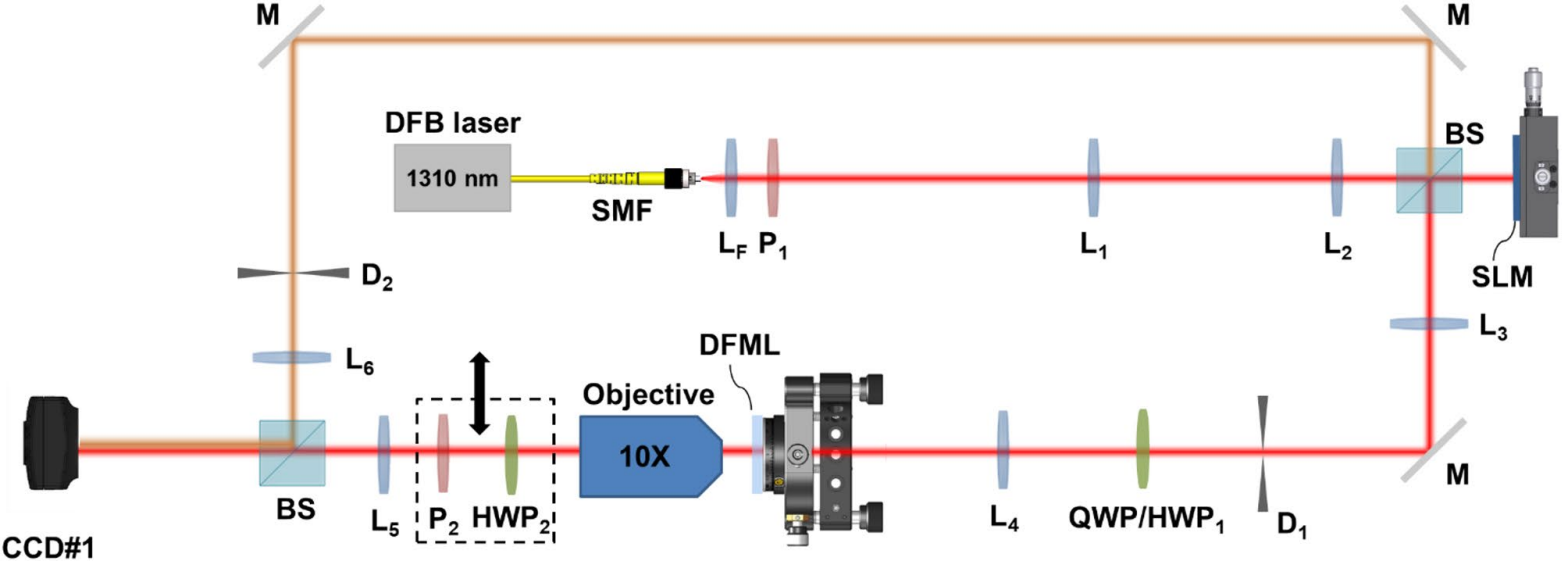
Layout of the experimental setup. The output of a DFB laser at 1310 nm is collimated (aspheric lens L_F_), linearly polarized (polarizer P_1_), and expanded (lenses L_1_, L_2_) before illuminating a LCoS SLM for beam shaping. A 50:50 beam-splitter (BS) is used to separate the input and reflected beam and create an interferometric arm (mirrors M, diaphragm D_2_, focusing lens L_6_). The resized Gaussian beam is filtered using a 4*f* setup (L_3_, L_4_, D_1_) and illuminates the sample (DFML) placed on a 6-axis kinematic mount. The input polarization state is controlled using either a quarter-wave plate (QWP) or a half-wave plate (HWP_1_). The output beam is expanded using a 10 × objective and an imaging lens (L_5_) and detected with a camera. A second half-wave plate can be inserted or removed to generated anti-vortex states. A rotating polarizer (P_2_) is used for vector beams analysis.

The optical behavior of the fabricated metalens has been tested using the experimental setup depicted in Fig. 9. The input Gaussian beam of the desired waist was generated with a (Liquid Crystal on Silicon) LCoS spatial light modulator (SLM) (X13267-08, Hamamatsu, pixel pitch 12.5 μm) using a phase and amplitude modulation technique^64^. The output of a DFB laser (λ = 1310 nm, 1310LD-1-2-2-1 CCSI, AeroDiode) was collimated at the end of a single mode fiber using an aspheric lens with focal length *f*_*F*_ = 7.5 mm (A375TM-C, Thorlabs), linearly polarized (LPIREA100-C, Thorlabs) and expanded by a first telescope (*f*_1_ = 3.5 cm, *f*_2_ = 10.0 cm) before illuminating the display of the SLM. Then, a 4-*f* system (*f*_3_ = 20.0 cm, *f*_4_ = 12.5 cm) with an aperture in the Fourier plane was used to isolate the first-order encoded mode. This optical architecture is ideal for amplitude/phase reshaping of the input beam and can be extended to further studies^65^. A 50:50 beam-splitter was placed before the SLM display to split the beam and use it in an interferometric arm. In between, a quarter-wave plate (QWP) (WPQ10M-1310, Thorlabs) or a half-wave plate (HWP_1_ in Fig. 9) (WPH05M-1310, Thorlabs) were used in order to set the desired polarization state. In the specific, the quarter-wave plate was exploited to generate circularly polarized states from the original horizontally polarized one, while the half-wave plate was used, alternatively, to rotate the polarization plane of the beam exiting the SLM and generate the desired vector beam.

The polarized Gaussian beam with the desired size illuminated the patterned zone of the DFML, mounted on a 6-axis kinematic mount (K6XS, Thorlabs). A 10 × Objective (CFI E Plan Achromat 10X, Nikon) mounted on a micrometric translator stage (LX20/M, Thorlabs) was used to both collect and focalize the generated beam. The removable sequence of a half-waveplate and a linear polarizer was used to select on demand the VB basis (HWP_2_ in Fig. 9) and to analyze the VB polarization configuration (P_2_), respectively. A relay lens (*f*_*5*_ = 7.5 cm) was used to image the collimated light after the objective into a camera (WiDy SWIR 640U-S, pixel pitch 12.5 µm). The different scale is related to the magnification factor introduced in the experimental setup. The presence of microscope objective and lens are necessary to extract and magnify properly the generated beams. As a matter of fact, the focal length of 500 μm is too short to collect the beam directly on the CCD camera. Moreover, the focused beam would be hardly appreciable due to the pixel size of the same order of the waist of the focused beam (see Fig. 5e,h). This explains the different scale bars between simulations and experimental acquisitions in Fig. 5. A second 50:50 beam-splitter was used to enable the interferometric arm on-demand (using an aperture) able to focalize (*f*_*6*_ = 12.5 cm) the input beam into the camera to detect the interferograms for the dual-functionality characterization (Fig. 5f,i).

## Supplementary Information

Supplementary Information.

## Data Availability

The original contributions presented in the study are included in the article, further inquiries can be directed to the corresponding author.
