# Supplementary material for: Dual-functional metalenses for the polarization-controlled generation of focalized vector beams in the telecom infrared
Source: Sci Rep. 2023 Jun 26;13:10327. doi: 10.1038/s41598-023-36865-z (PMC10293257; doi:10.1038/s41598-023-36865-z)
Supplement: Supplementary file 1 — Supplementary Information. [file 41598_2023_36865_MOESM1_ESM.pdf]

SUPPLEMENTARY MATERIAL

Dual-functional metalenses for the polarization-controlled generation of focalized vector beams in the telecom infrared

Andrea Vogliardi[1,2,†], Gianluca Ruffato[1,2,†,*], Simone Dal Zilio[3], Daniele Bonaldo[1], and Filippo Romanato[1,2,3]

[1] Department of Physics and Astronomy 'G. Galilei', University of Padova, via Marzolo 8, 35131 Padova, Italy

[2] Padua Quantum Technologies Research Center, University of Padova, via Gradenigo 6, 35127 Padova, Italy

[3] CNR-IOM Istituto Officina dei Materiali, S.S. 14 - Km. 163,5 - 34149 Trieste (TS), Italy

[†]The two authors contributed equally

***Correspondence:** gianluca.ruffato@unipd.it

Keywords: vector beams, metalens, dual function, metasurface, infrared, silicon

S1. GENERATION OF HIGH ORDER VECTOR BEAMS

For the benefit of the reader, we provide here some examples to prove that our approach can be used for generation of different kinds of complex vector beams. In particular, we apply the same library of 13 silicon pillars shown in Fig. 3 of the main manuscript, optimized for the working wavelength of 1310 nm, to design dual-functional metasurface for the generation of high-order OAM beams.

First of all, we show the generation of 5th order vector beams with azimuthal index ℓ=±5 and radial index p=0, using the design formula $\phi^{\pm} = \phi_{LG}^{\pm 5} + \phi_f$, where $\phi_{LG}^{\pm 5} = \pm i5\varphi$ and ϕ_f is a polarization-insensitive spherical term for focusing on the optical axis at a distance f from the optical element. From the theory we obtain the spin-decoupled scalar basis generation for impinging circularly polarized states (Figure S1):

$$J\left|L\right\rangle = e^{i\left(\phi_{LG}^{+5}+\phi_f\right)}\left|R\right\rangle \quad (1)$$

$$J\left|R\right\rangle = e^{i\left(\phi_{LG}^{-5}+\phi_f\right)}\left|L\right\rangle \quad (2)$$

and the vector beams generation by the illumination with arbitrary linearly-polarized light:

$$J\left|\theta\right\rangle = e^{i\phi_f}\left(e^{i\phi_{LG}^{+5}}e^{-i\theta}\left|L\right\rangle + e^{i\phi_{LG}^{-5}}e^{+i\theta}\left|R\right\rangle\right) \quad (3)$$

It must be considered that either switching the phase profiles carried by the two circular polarizations or flipping the chirality of the output polarization state, *e.g.*, using a half-wave plate, it is possible to generate the complementary vector bases (anti-vortices):

$$J\left|\theta\right\rangle = e^{i\phi_f}\left(e^{i\phi_{LG}^{+5}}e^{-i\theta}\left|R\right\rangle + e^{i\phi_{LG}^{-5}}e^{+i\theta}\left|L\right\rangle\right) \quad (4)$$

As depicted in Figure S2, the simulated intensity and phase profiles are consistent with the theoretical behaviour expected by Eq. (3) and (4), under the filtering action of a rotating linear polarizer.

Then, we show the possibility to generate vector beams which have also radial discontinuities, *i.e.,* a radial index p>1. From the literature, it is suggested that the phase function of a phase-only element able to generate high-order LG beams is:

$$U_p^{\ell}(r,\varphi) = e^{i\ell\varphi} \cdot sign\left[L_p^{|\ell|}\left(\frac{2r^2}{w_0{}^2}\right)\right] \tag{5}$$

being ℓ and p the azimuthal and radial indices, respectively, $L_p^{|\ell|}$ the associated Laguerre polynomial, and w_0 the input beam waist [1] . For the sake of simplicity, we define $c_p^{|\ell|} = sign\left[L_p^{|\ell|}\left(2r^2 / w_0{}^2\right)\right]$, then Eq. (1)-(3) can be generalized as:

$$J\left|L\right\rangle = c_p^{|\ell|} e^{i\left(\phi_{LG}^{+\ell}+\phi_f\right)} \left|R\right\rangle \tag{6}$$

$$J\left|R\right\rangle = c_p^{|\ell|} e^{i\left(\phi_{LG}^{-\ell}+\phi_f\right)} \left|L\right\rangle \tag{7}$$

$$J\left|\theta\right\rangle = c_p^{|\ell|} e^{i\phi_f}\left(e^{i\phi_{LG}^{+\ell}} e^{-i\theta}\left|L\right\rangle + e^{i\phi_{LG}^{-\ell}} e^{+i\theta}\left|R\right\rangle\right) \tag{8}$$

obtaining a set of equations describing the generation of high-order scalar and vector beams with non-null radial index. Again, it is worth noting that it is possible to generate also the complementary basis:

$$J\left|\theta\right\rangle = c_p^{|\ell|} e^{i\phi_f}\left(e^{i\phi_{LG}^{+\ell}} e^{-i\theta}\left|R\right\rangle + e^{i\phi_{LG}^{-\ell}} e^{+i\theta}\left|L\right\rangle\right) \tag{9}$$

using the abovementioned techniques. In Figure S3 and Figure S4, the spin-decoupled generation of scalar beams using circularly polarized input beams and the production of the corresponding vector beams under linearly polarized illumination are shown, respectively.

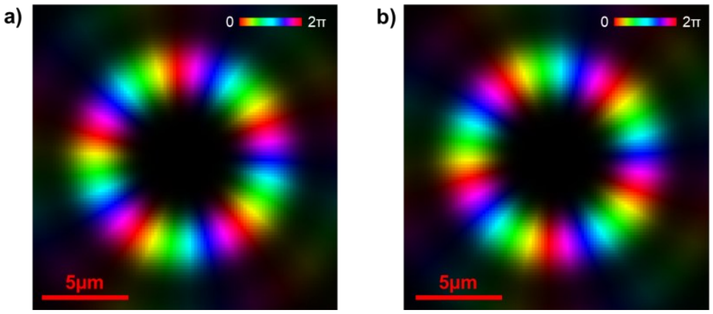

Figure S1: Generation of 5*th* order scalar OAM beams using spin-decoupled metasurface with the design formulas in Eqs. (1) and (2), for λ = 1310 nm, using the silicon metaunits in Fig. 3 of the main manuscript. (a) Simulated intensity and phase of the propagated field under RCP polarization at z = 500 µm. (b) Simulated intensity and phase of the propagated field under LCP polarization at z = 500 µm. Brightness and colors refer to intensity and phase, respectively. It can be noticed that the generated OAM beams exhibit the same intensity distributions but opposite azimuthal helicity (*i.e.*, $\ell = \pm 5$).

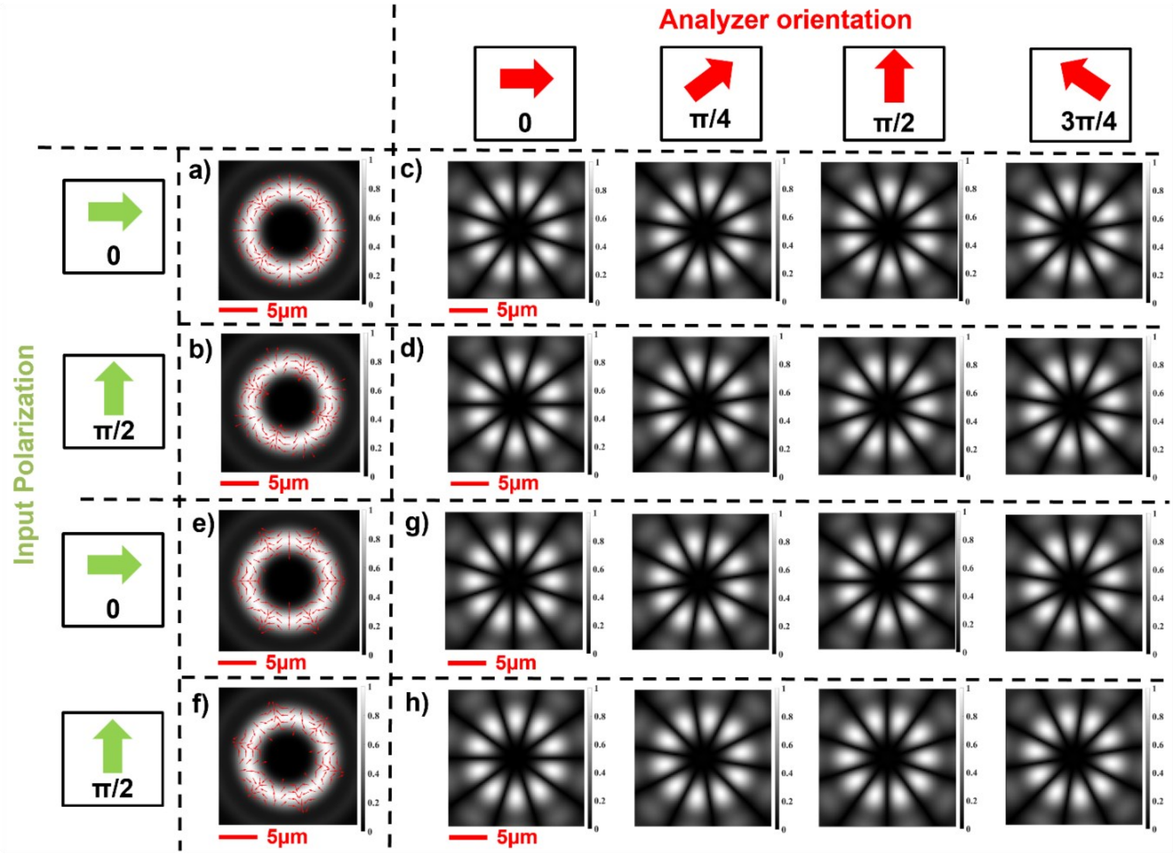

Figure S2: Generation of 5th order vector beams following the design criteria of Eqs. (3) and (4) and analysis using a rotating linear polarizer (LP). (a-d) Simulations referring to the design recipe using Eq. (3) for vortex states. (a) Simulated intensity profile and polarization plot of the generated VB using impinging horizontally polarized light. (b) Simulated intensity profile and polarization plot of the generated VB using impinging vertically polarized light. (c)-(d) Intensity profiles of the vector beam analyzed with a rotating LP. (e-h) Simulations referring to the design recipe using Eq. (4) for anti-vortex states. (e) Simulated intensity profile and polarization plot of the generated VB using impinging horizontally polarized light. (f) Simulated intensity profile and polarization plot of the generated VB using impinging vertically polarized light. (g)-(h) Intensity profiles of the anti-vortex vector beams analyzed with a rotating LP.

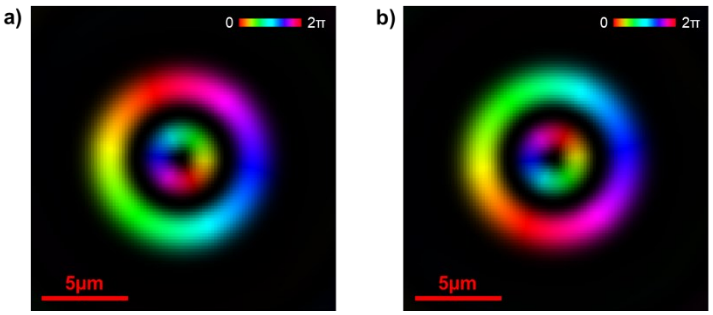

Figure S3: Generation of scalar OAM beams (ℓ=±1) with non-null radial index (p=1) using the design formulas in Eqs. (6) and (7), for λ = 1310 nm, using the silicon metaunits in Fig. 3 of the main manuscript. (a) Simulated intensity and phase of the propagated field under RCP polarization at z = 500 µm. (b) Simulated intensity and phase of the propagated field under LCP polarization *at* z = 500 µm. Brightness and colors refer to intensity and phase, respectively. It can be noticed that the generated OAM beams exhibit the same intensity profile but opposite azimuthal helicity.

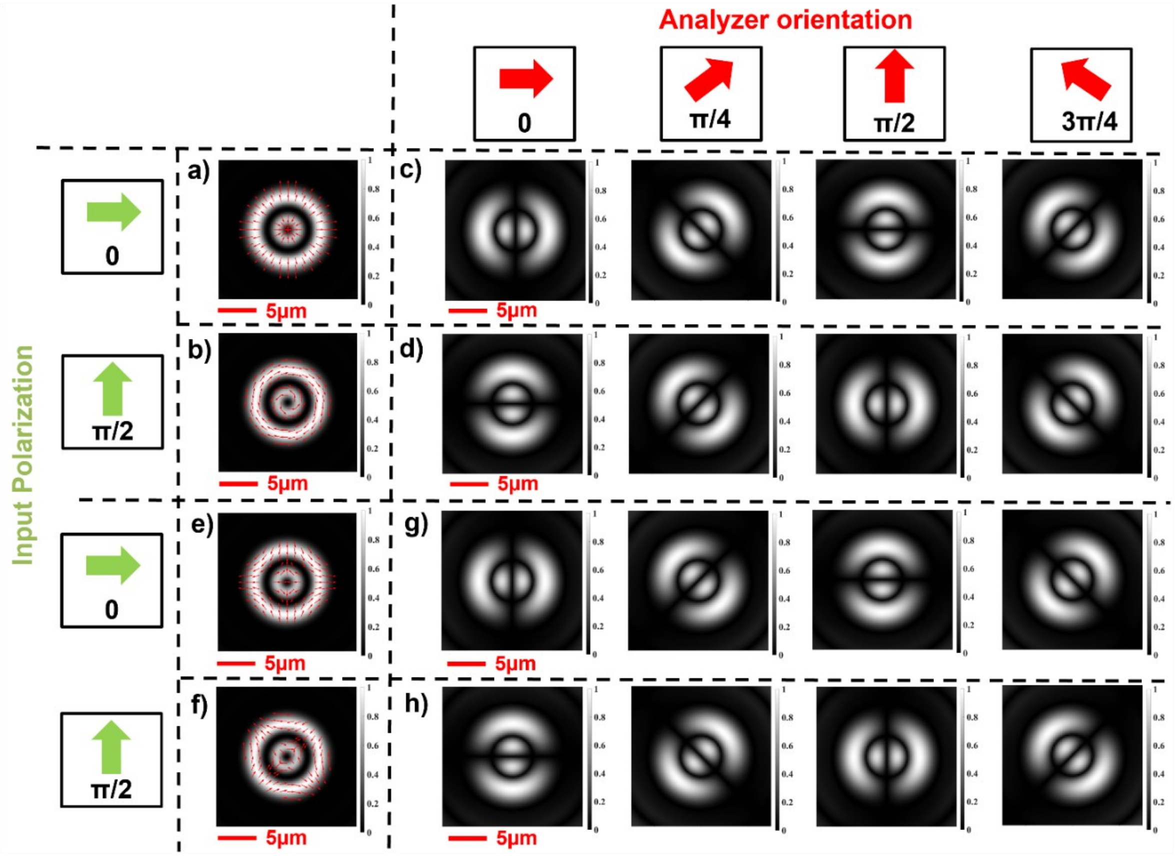

Figure S4: Generation of vector beams with non-null radial index following the design criteria of Eqs. (8) and (9) and analysis using a rotating linear polarizer (LP). (a-d) Simulations referring to the design recipe using Eq. (8). (a) Simulated intensity profile and polarization plot of the generated VB using impinging horizontally polarized light. (b) Simulated intensity profile and polarization plot of the generated VB using impinging vertically polarized light. (c)-(d) Intensity profiles of the vector beams analyzed with a rotating LP. (e-h) Simulations referring to the design criteria using Eq. (9) for anti-vortex states. (e) Simulated intensity profile and polarization plot of the generated VB using impinging horizontally polarized light. (f) Simulated intensity profile and polarization plot of the generated VB using impinging vertically polarized light. (g)-(h) Intensity profiles of the anti-vortex vector beams analyzed with a rotating LP.

S2. METAOPTICS ROBUSTNESS FOR METAATOMS HEIGHT DEVIATION

For the benefit of the reader, we recall the Jones matrix of each metaatom (omitting the spatial dependance to simplify the notation):

$$J = e^{i\frac{\delta_x+\delta_y}{2}} \cos\left(\frac{\Delta}{2}\right)\begin{bmatrix} 1 & 0 \\ 0 & 1 \end{bmatrix} - ie^{i\frac{\delta_x+\delta_y}{2}} \sin\left(\frac{\Delta}{2}\right)\begin{bmatrix} \cos(2\theta) & \sin(2\theta) \\ \sin(2\theta) & -\cos(2\theta) \end{bmatrix} \tag{10}$$

being θ the local orientation of the metaatom fast-axis and $\Delta = \delta_y - \delta_x$ the phase retardation between the two axes of the metaunit.

Under the choice $\Delta = \pi$ the optical behaviour of the metaunit is that of a rotated half-wave plate, therefore, for circularly-polarized light in input, each metaatom behaves as a polarization converter, as assumed in Eqs (1)-(2) and Eqs. (6)-(7). On the contrary, a deviation from the half-wave plate condition is expected to introduce a component which has the same circular polarization of the incident light, the so-called zero-order contribution. Moreover, it must be considered that all these conditions are valid if each metaatom has similar transmissions for *TE* and *TM* polarizations [2] [3] .

In the following, we studied the different performances of our metaoptics depending on the deviation from the ideal design in terms of pillars height. In particular, it has been simulated both the transmission and the phase delay (Figure S5 and S6) under *TE* and *TM* polarization for all the types of pillars sweeping different heights within a range centered on the optimal height, *i.e.*, 850 nm, and a maximum deviation of 50 nm, which is far greater than the experimental accuracy of the etching technique. Thus, the $\sin^2(\Delta/2)$ parameter has been calculated and considered in order to estimate the polarization conversion efficiency of each metaunit in such different conditions.

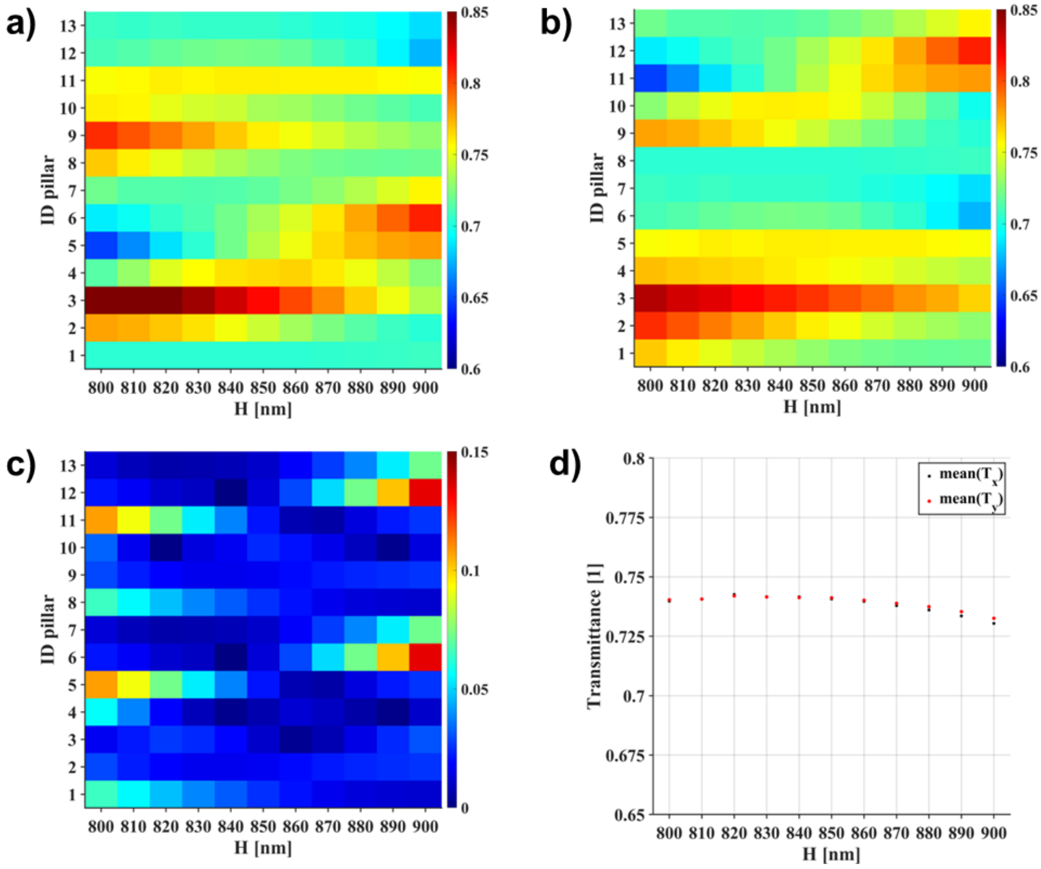

Figure S5: Simulation of the transmittance variation for different pillars height in the range between 800 and 900 nm, step 10 nm. The pillars number (1-13) refers to the set in Fig. 3 of the main manuscript, showing the set of silicon metaunits optimized for the thickness of 850 nm. (a) T_x transmittance, (b) T_y transmittance, and (c) difference $|T_x$-$T_y|$ for each type of metaunit as a function of the pillars height. (d) Metalens average trasmittances for several pillars heights.

Figure S5(a-b) shows, as expected, some variations in terms of transmittance in concurrence with the height change, but it is significant to notice that the difference between T_x and T_y is below 0.075 in the range from H = 820 nm to H = 880 nm (Figure S5(c)). Moreover, for higher deviations only few pillars have higher transmission difference between T_x and T_y, while on average the difference is still limited (Figure S5(d)).

A similar behavior can be observed in terms of phase difference (Δ) between *TE* and *TM* polarizations (*i.e.,* δ_y and δ_x), in fact, for H in the range between 820 nm and 880 nm, Δ has a

variation between 2.9 and 3.4, corresponding to a $\sin^2(\Delta/2)$ higher than 0.98 for all the pillars. However, analyzing the global $\sin^2(\Delta/2)$, calculated as the average of all the 13 metaunits, it can be noticed that the polarization efficiency is above 0.99 within the whole interval from 800 to 900 nm (Figure S6). In conclusion, it is possible to state that the designed metasurface is robust in terms of transmission and polarization conversion efficiency in the considered range between 800 and 900 nm.

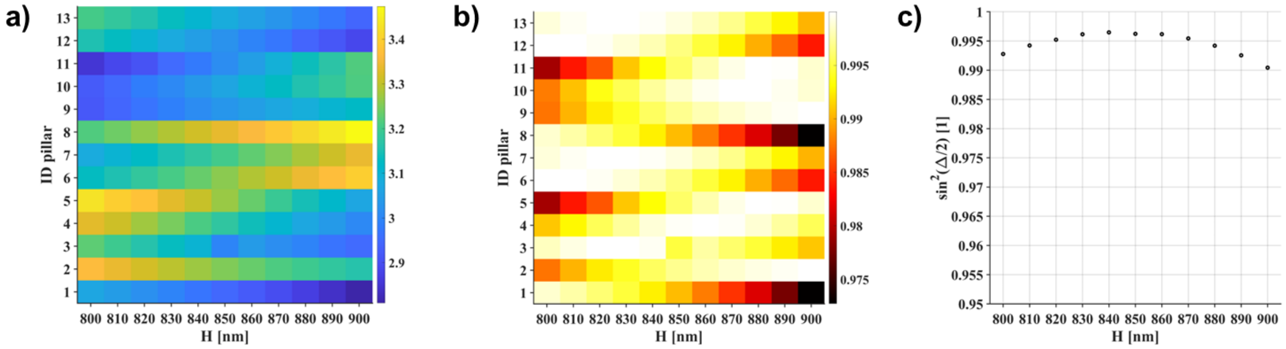

Figure S6: (a) Phase difference (Δ) between TE and TM polarizations (δ_y and δ_x), and (b) conversion efficiency $\sin^2(\Delta/2)$ for each type of metaunit with different heights. The pillars number (1-13) refers to the set in Fig. 3 of the main manuscript, showing the set of silicon metaunits optimized for the thickness of 850 nm. Working wavelength: 1310 nm. (c) Metalens average $\sin^2(\Delta/2)$ for different pillars height.

S3. BROADBAND METAOPTICS PERFORMANCE

In order to test the performance of the designed metaoptics within a broad wavelength range, we used the same approach described in section **S2**. Thus, it has been simulated both the transmissions and the phase delays under *TE* and *TM* polarization for all the types of pillars sweeping different wavelengths within the whole telecom O-band (1260-1360 nm). Then, the $\sin^2(\Delta/2)$ parameter has been calculated in order to estimate the polarization conversion efficiency of the metaoptics under impinging wavelengths which are different from the optimal one, *i.e.*, λ = 1310 nm.

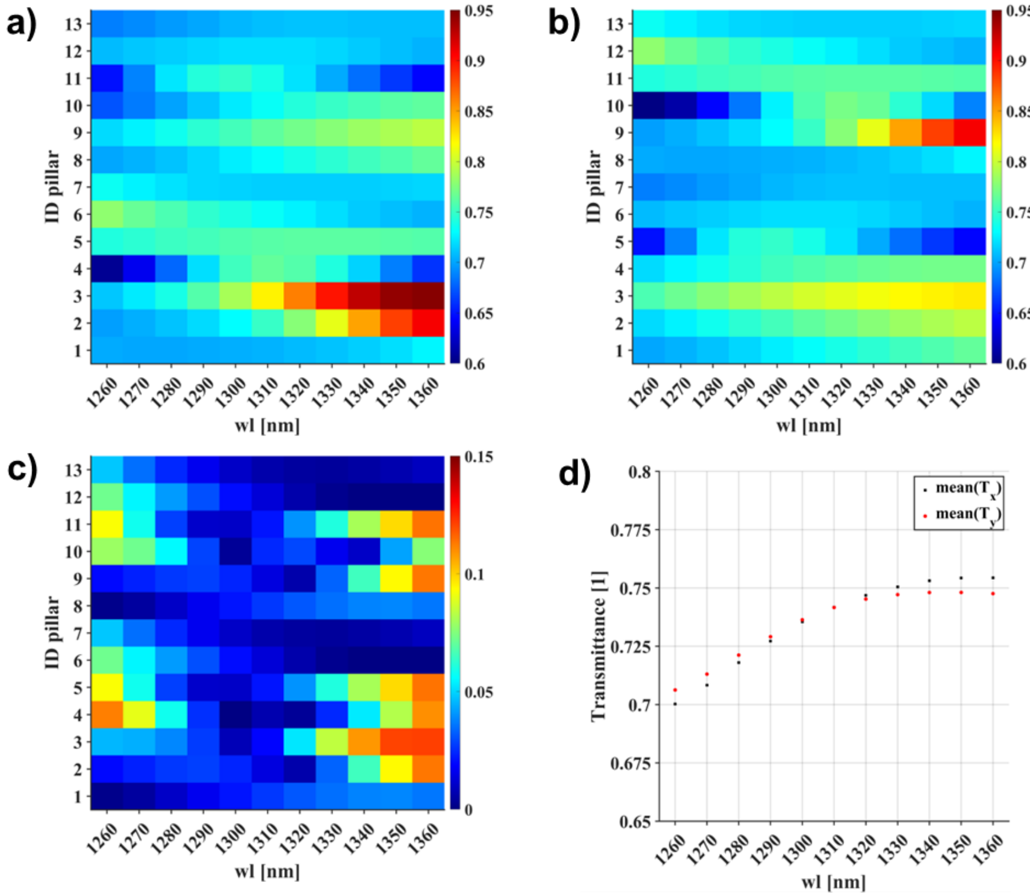

Figure S7: Simulation of the transmittance variation for different illuminating wavelengths in the range between 1260 and 1360 nm, step 10 nm. (a) T_x transmittance, (b) T_y transmittance, and (c) difference $|T_x$-$T_y|$ for each type of metaunit. The pillars number (1-13) refers to the set in Fig. 3 of the main manuscript, showing the library of silicon metaunits optimized for the wavelength of 1310 nm. (d) Metalens average trasmittance as a function of the illuminating wavelength.

Figure S7(a-b) shows, as expected, some variations in terms of transmission associated to a wavelength sweeping. Moreover, it is worth noting that the difference between T_x and T_y is under 0.075 in the range from λ=1280 nm to λ=1330 nm (Figure S7). Unlike the height deviations study, in this case it can be observed that outside the abovementioned range many pillars exhibit a high transmission difference between *TE* and *TM* polarization. To confirm these considerations, in Figure S7 it can be noticed that, on average, the overall difference $|T_x$-$T_y|$ increases more significantly as the wavelength falls outside the interval 1280-1330 nm. Concurrently, from Figure S8(b) it turns out that in the band between λ= 1290 nm and 1330 nm the metaoptics have locally a polarization conversion

efficiency higher than 0.98. Then, considering the global polarization conversion parameter, this band could be extended to the range from λ=1280 nm to1350 nm. In the whole 100 nm band 1260 nm and 1360 nm (telecom O-band) the metasurfaces are expected to show a zero-order contribution lower than 5%.

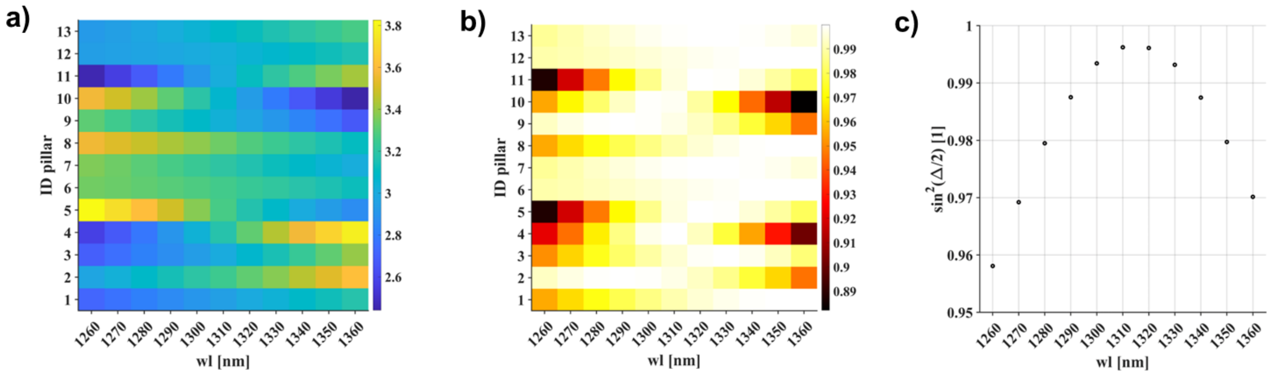

Figure S8: (a) Phase difference (Δ) between *TE* and *TM* polarizations (δ_y and δ_x), and (b) conversion efficiency for each type of metaunit with different illuminating wavelengths. (c) Metalens average conversion efficiency as a function of the illuminating wavelength.

To confirm the broadband behavior of the metaoptics we simulated the illumination of a metasurface, designed to generate a first order basis at a focal length of 500 µm, with circularly polarized light in input at different wavelengths in the telecom O-band (λ= 1260-1360 nm). As depicted in Figure S9, the simulated focal length of the generated beams shifts depending on the input wavelength following the theoretical trend. Therefore, while the metalens is not achromatic, on the other hand it focuses light at the expected position and maintaining a high efficiency. As discussed in the main text, by further engineering the group delay and group delay dispersion of the metaatoms, the phase change induced by a different wavelength could be compensated in order to make the focusing operation achromatic.

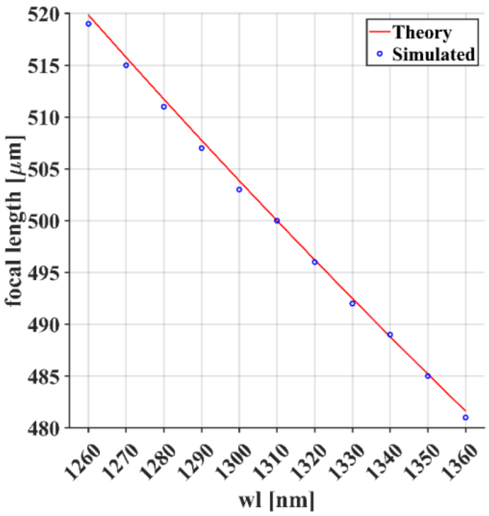

Figure S9: Simulated focal length position as a function of the illuminating wavelengths, and comparison with the theoretical trend.

S4. PURITY OF THE GENERATED OAM MODES

The purity of the OAM beams produced by the designed metasurfaces has been estimated numerically by analysing the OAM spectrum of the generated beam $U(\rho,\theta)$ at the focal plane using the expression [4] :

$$\eta_l = \frac{I_l}{I} = \frac{\int_0^{+\infty} |u_l(\rho)|^2 \rho d\rho}{\sum_{l=-\infty}^{+\infty} \int_0^{+\infty} |u_l(\rho)|^2 \rho d\rho} \quad (11)$$

where $u_l(\rho) = \frac{1}{2\pi} \int_{-\pi}^{+\pi} U(\rho,\theta) \exp(-il\theta) d\theta$.

In Figure S10 we report the OAM spectrum analysis of a first order vortex state generation by a linearly polarized light for different height of the metaatoms. The purity of the generated OAM beams shows no significative difference for the two outgoing circularly polarized beams. It reaches the maximus at *H*= 850 nm and it is still higher than 0.99 for a deviation of 20 nm from the optimal thickness (*i.e.*, for *H* = 830 nm and *H* = 870 nm). The purity decreases slightly when the height

differs by 50 nm from the designed one (0.987 for H=800 nm, and 0.984 for H=900 nm). So, as previously suggested by the analysis performed in section S2, we verify that the conversion efficiency is very high for all the different conditions.

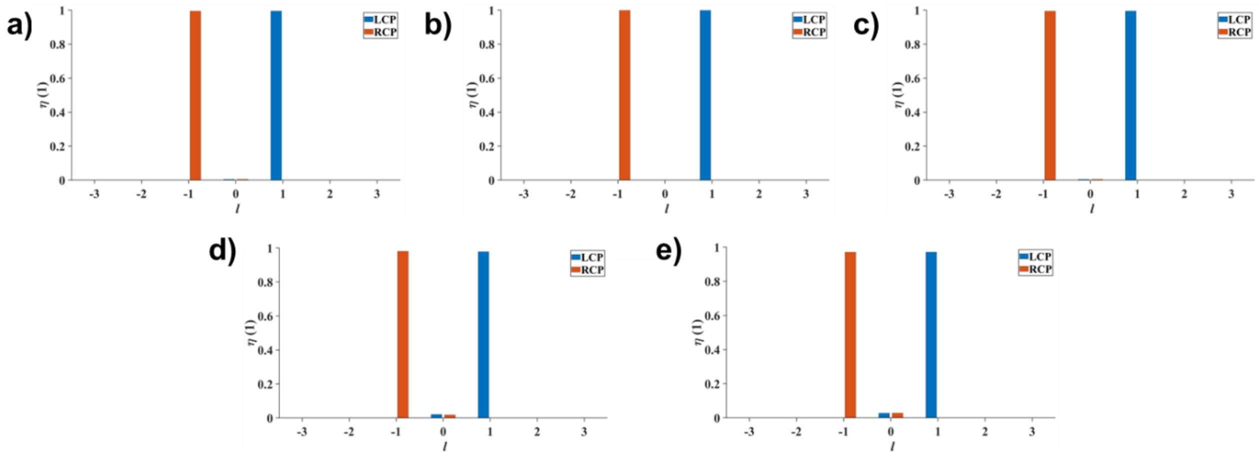

Figure S10: OAM spectrum analysis of a first order vortex state generation by a linearly polarized light for different metaatoms height: (a) H = 830 nm, (b) H = 850 nm, and (c) H =870nm. In all these cases the OAM purity is above 0.99. In (d) H = 800 nm and (e) H = 900 nm the OAM purity decreases because of the increasing of the zero-order contribution due to the condition $\Delta \neq \pi$.

In Figure S11 we show the OAM spectrum analysis of a first order vortex state generation by a horizontally polarized light at different wavelengths. Also in this case the purity of the generated OAM beams shows no significative difference for the two outgoing circularly polarized beams. The purity decreases as soon as the illumination wavelength differs from the designed one, in particular, it decreases from 0.998 at λ=1310nm to 0.947 at λ= 1260 nm therefore, a purity of the generated OAM higher than 0.94 over the whole band supports the statements made in section S3, claiming that the metaoptics exhibits a broadband behaviour between λ= 1260 nm and λ= 1360nm, *i.e.,* within the whole telecom O-band.

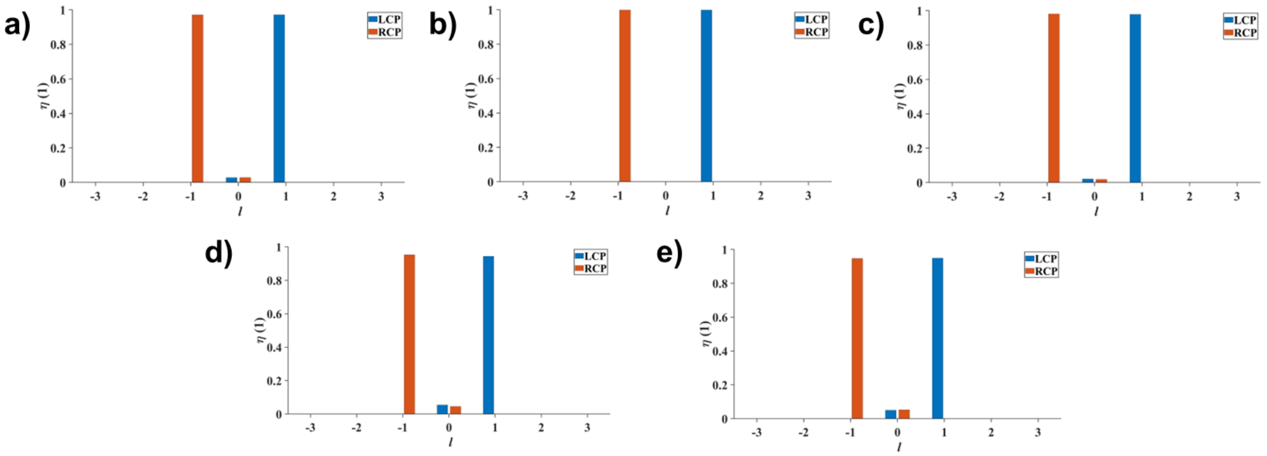

Figure S11: OAM spectrum analysis of a first order vortex state generation impinged by a linearly polarized light for different illuminating wavelengths. (a) λ = 1290 nm, (b) λ = 1310 nm, and (c) λ = 1330 nm. In all these cases the OAM purity is above 0.98. In (d) λ = 1260 nm and (e) λ = 1360 nm the OAM purity drops down to around 0.94 because of the increasing of the zero-order contribution due to $\Delta \neq \pi$.

REFERENCES

[1] Ruffato, G., Massari, M., & Romanato, F. Generation of high-order Laguerre–Gaussian modes by means of spiral phase plates. *Optics letters*, **39**(17), 5094-5097 (2014).

[2] Vogliardi, A., Romanato, F. & Ruffato, G. Design of Dual-Functional Metaoptics for the Spin-Controlled Generation of Orbital Angular Momentum Beams. *Front. Phys.* **586** (2022).

[3] Ruffato, G. & Romanato, F. Design of continuously variant metasurfaces for conformal transformation optics. *Opt. Express* **28** (23), 34201-34218 (2020).

[4] Ruffato, G., Massari, M., & Romanato, F. Multiplication and division of the orbital angular momentum of light with diffractive transformation optics. *Light: Science & Applications*, **8**(1), 113 (2019).
